# Unveiling Diet Preferences and Their Nutritional Drivers Through Metabarcoding: The Case of Alpine Marmot (*Marmota marmota* L.) in High Altitude Grazed Pastures

**DOI:** 10.1002/ece3.73309

**Published:** 2026-03-22

**Authors:** Giorgio Marchesini, Elena Basso, Cristina Pornaro, Piergiovanni Partel, Gilberto Volcan, Enrico Dorigatti, Rudi Cassini, Erica Marchiori, Salvatore Raniolo, Massimo Pindo, Andrea Squartini

**Affiliations:** ^1^ Department of Animal Medicine Production and Health University of Padova Padova Italy; ^2^ Department of Agronomy, Food, Natural Resources Animals and Environment University of Padova Padova Italy; ^3^ Paneveggio Pale di San Martino Natural Park Trento Italy; ^4^ Research and Innovation Centre Fondazione Edmund Mach San Michele all'Adige Italy

**Keywords:** diet richness, food selection, Hill numbers, nutrient composition, pasture quality, rodent

## Abstract

This study employed DNA metabarcoding to investigate the diet composition and foraging strategies of alpine marmots ( *Marmota marmota* L.) across diverse alpine pasture ecosystems throughout the summer season. Fecal samples from nine family units in four distinct areas were analyzed, alongside comprehensive pasture floristic and nutritional assessments. The study identified 86 plant species as typical in the marmot diet, revealing a more diverse dietary repertoire than previously reported. Diet composition varied across locations and throughout the summer, with differences not strictly linked to plant availability. Notably, the observed correlation between the abundance of specific plant species in the diet and overall pasture nutritional quality, coupled with the differential selection (positive or negative) of certain genera, suggests that marmots employ adaptive foraging strategies in response to fluctuations in nutrient availability. Factors such as protein content, fiber levels, and fatty acid profiles influenced plant selection. Additionally, cattle grazing appeared to impact marmot diet composition, likely through its effects on plant phenology and nutrient content. The study highlights the complex interplay between plant availability, nutritional composition, and environmental factors in shaping marmot foraging behavior. These findings contribute to our understanding of alpine marmot ecology and have implications for pasture management strategies in alpine ecosystems.

## Graphical abstract

The alpine marmot ( *Marmota marmota* ), a crucial component of alpine ecosystems, serves both as a key player in the food chain and as an ‘ecosystem engineer.’ Employing DNA metabarcoding of fecal samples, this study investigated the diet composition and foraging strategies of four alpine marmot colonies across diverse alpine pasture ecosystems throughout the summer season. Results reveal a diverse diet comprising at least 86 plant species, with significant variations across locations and time, influenced by protein content, fiber levels, and fatty acid profiles of pastures, as well as cattle grazing proxies.

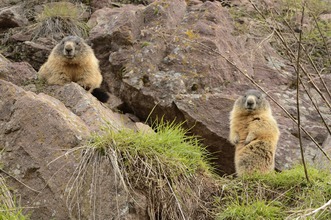

## 1 Introduction

Herbivores are often perceived as being limited by food resources (White 2008), as their diet significantly defines their survival prospects, behavior, and ecological interactions. A deficiency in either the quality or quantity of available forage may have serious drawbacks such as the reduction of population size driven by the impact on some demographic parameters including age at maturity, last age of reproduction, juvenile survival, adult survival, and fertility (Dobson and Oli 2001; White 2008). Consequently, feeding behaviors that allow animals to procure sufficient high‐quality food resources are assumed to be strongly selected (Goldberg et al. 2020). Therefore, elucidating the dietary range and preferred food items of a species is crucial for comprehending its ecological niche and adaptive strategies (Belovsky 1986).

Most mammalian herbivores consume a diverse array of plants and may exhibit preferences for certain plants because of differences in nutrient profiles across food items (Castle et al. 2020) or to regulate the ingestion of various plant secondary compounds (Torregrossa et al. 2011). Although generalist herbivores consume many different plants and plant parts (seeds, roots, and leaves), they do not necessarily consume everything encountered in proportion to its availability (Aryal et al. 2015; Goldberg et al. 2020). Small and medium‐sized herbivores are particularly inclined to be selective, as their short digestive tracts limit intake volumes. Hence, these small herbivores often face trade‐offs between coping with plant defenses, minimizing search times, and maximizing nutrient intake per unit consumed (Belovsky 1986). Furthermore, these foraging trade‐offs likely fluctuate seasonally as plant defenses, food availability, and nutritional requirements change (Soininen et al. 2013). Even though experimental studies demonstrate that the availability of a high‐quality food item can reduce the consumption of a low‐quality food item, in natural environments, the proportion of any preferred food item in diets would increase with its availability but would also be affected by the availability of alternative food items (Soininen et al. 2013).

Hibernating animals may be especially sensitive to food quantity and quality changes, as their nutritional needs may differ from those of non‐hibernators, and these nutrients must be acquired within a relatively brief annual window. Many hibernating mammals seasonally exhibit hyperphagia and alter their diets, often switching to plants or plant parts rich in specific polyunsaturated fatty acids (e.g., seeds) prior to hibernation, boosting overwinter survival (Lehmer et al. 2006; Ruf and Arnold 2008). Documenting seasonal shifts in preferred forage is thus crucial for hibernating herbivores, necessitating diet studies with sufficient taxonomic resolution to detect species‐level changes in favored food items.

The alpine marmot ( *Marmota marmota* L.), a medium‐sized hibernating social rodent that inhabits high‐altitude open areas of Western, Central, and Eastern Europe, plays an important role in the alpine ecosystem both as part of the food chain and as an “ecosystem engineer” (Forti et al. 2025). Marmots, in fact, modify the physical and chemical properties of the soil through their burrowing activity (Chibowski et al. 2023) and influence vegetation patterns through foraging. Alpine marmots exhibit a highly synchronized annual life cycle, characterized by a short period of above‐ground activity. Their reproductive phase commences promptly following female emergence from hibernation, typically occurring from late April to June (Exner et al. 2003). This species is characterized by a single annual reproductive event, with individuals returning to their hibernacula between September and October. Given the constrained duration of their active season, spanning approximately 4–5 months, alpine marmots face significant time pressure to mate, gestate, birth, and rear offspring, whereas simultaneously accumulating sufficient body mass to sustain them through the impending hibernation period (Armitage 2012). This temporal constraint imposes a strong selective pressure for efficient foraging strategies. Consequently, it is hypothesized that alpine marmots have evolved to preferentially target and consume the most nutritionally advantageous food items available within their habitat. This selective feeding behavior is likely crucial for optimizing energy intake and physiological preparation for the challenges of reproduction and extended hibernation.

Understanding the role of environmental and anthropogenic factors affecting the alpine marmot, particularly those that may compromise the quality and availability of pastures or influence the feeding activity of this species (Mainini et al. 1993; Uchida and Blumstein 2021), remains crucial for its conservation. For this reason, comprehensive baseline data on the marmots' dietary composition are crucial to assess how habitat management practices influence preferred forage plants and to develop optimal strategies for preserving their habitat.

Alpine marmots are known to exhibit a diverse diet (Bassano et al. 1996; Massemin et al. 1996; Rudatis and Battisti 2006; Garin et al. 2008), consisting of a wide variety of plant parts such as leaves, flowers, and fruits of a few plant families, classifying them as generalist foragers. However, previous studies have focused on a few locations in France (Massemin et al. 1996), Pyrenees (Garin et al. 2008) and two areas of the Italian Alps (Bassano et al. 1996; Rudatis and Battisti 2006), have employed micro histological techniques, which in some cases have shown limited taxonomic resolution (Soininen et al. 2009), did not consider plant availability in the pasture (Bassano et al. 1996; Massemin et al. 1996; Rudatis and Battisti 2006) and did not investigate the drivers of plant selection.

To enhance the understanding of diet breadth and optimal forage for this species, the following questions were addressed: (a) How diverse is the alpine marmot's diet, and to what extent do marmots selectively forage on specific plant species relative to their availability in the habitat?

(b) How does the alpine marmot's diet vary across different locations and throughout the summer season? (c) What is the relationship between the alpine marmot's diet and plant nutrient composition, and what other potential factors influence their dietary choices? Elucidating whether and how diet varies seasonally would aid in determining which plants are important to make alpine marmot populations thrive.

## 2 Materials and Methods

### 2.1 Study Area and Animals

**FIGURE 1:**
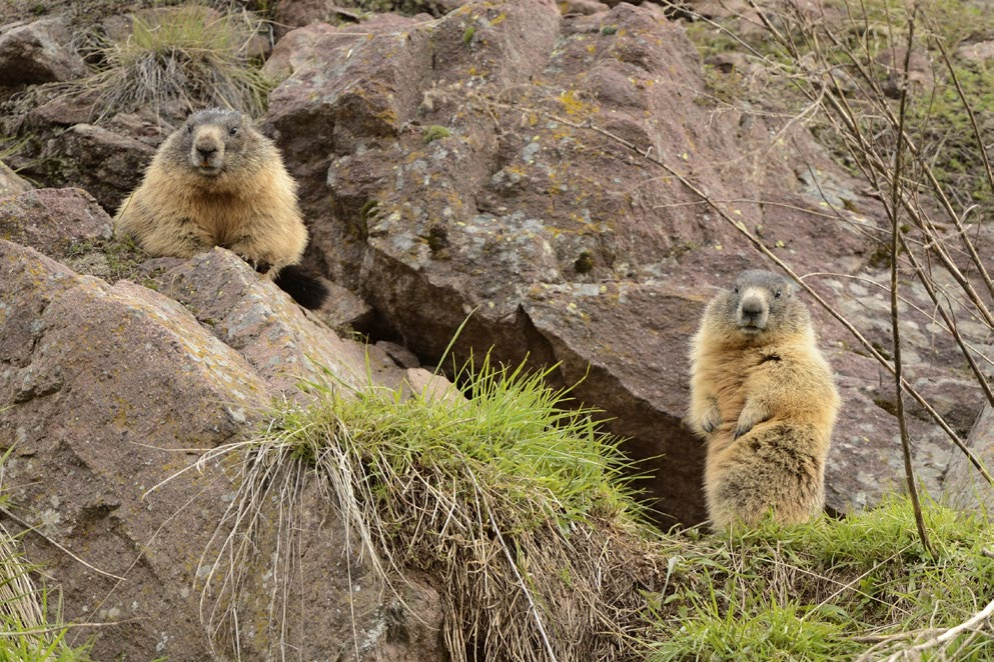
Photograph of Alpine marmots in the study area, authored by Bruno Bressan.

**FIGURE 2:**
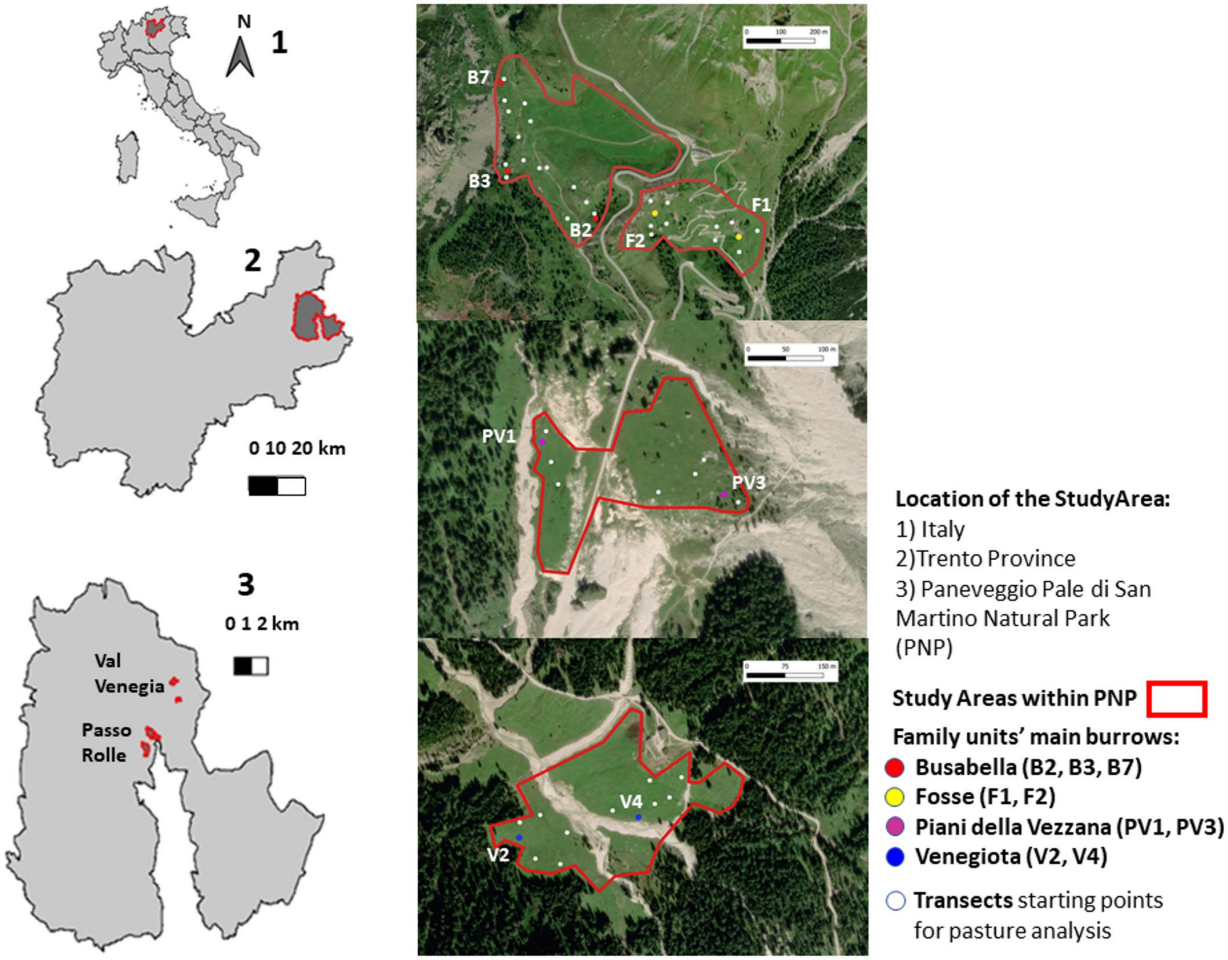
Map of the study area during the summer 2022. The areas studied are Busabella and Fosse, Piani della Vezzana, and Venegiota. For each family unit's main burrow (B2, B3, B7, F1, F2, PV1, PV3, V2, and V4), the starting points of all the linear transects used to collect herbage samples and perform floristic analysis are present.

In the current study, a comprehensive sampling of alpine marmot (Figure 1) fecal pellets throughout the summer of 2022 was conducted. The research encompassed nine family units distributed across four distinct alpine pasture zones at varying elevations within the Paneveggio Pale di San Martino Natural Park (PNP) in north‐eastern Italy (Figure 2).

The study sites comprised two macro‐areas: Passo Rolle (46° 17′ N, 11° 47′ E) and Val Venegia (46° 31′ N, 11° 49′ E). Passo Rolle included the areas of Busabella (B) and Fosse (F), where three and two family units were studied, respectively. These areas, situated at approximately 1900 m a.s.l. (range: 1830–1984 m), are characterized by subalpine grasslands bordered by natural barriers and interspersed with a timberline ecotone formed by sapling establishment of Norway spruce ( *Picea abies* ) and European larch ( *Larix decidua* ).

The other two areas in Val Venegia, Venegiota (V) and Piani della Vezzana (PV), where for each two family units were studied, are located at elevations ranging from 1780 to 1960 m a.s.l. Venegiota, adjacent to Malga Venegiota (1820 m a.s.l.), is predominantly surrounded by coniferous forests and features herbaceous vegetation intersected by a small, gravel‐lined stream. Both areas are utilized for cattle grazing and host a small population of donkeys. Piani della Vezzana exhibits a notably homogeneous and level topography, with herbaceous cover interspersed with isolated trees and numerous rock formations of varying sizes. In contrast to the more undulating terrain of Busabella and Fosse, both Venegiota and Piani della Vezzana present more uniform landscapes. The climate across all study areas is characterized by mild summers and severe winters, with snow cover persisting for 6–7 months annually, typically from late November to half May.

The estimated number of individuals in the different marmot's family units in June 2022 is reported in Table 1.

**TABLE 1:** Estimated number of alpine marmot individuals in different family units in June 2022. ND, not determined.

Macroarea	Area	Family unit	Tot individuals, n	Adults, n	Subadults, n	Juveniles	Pups
Passo Rolle	Busabella	B2	11	2	0	4	5
		B3	2	2	0	0	0
		B7	6	1	5	0	0
	Fosse	F1	5	1	0	4	0
		F2	5	2	0	0	3
Val Venegia	Piani della Vezzana	PV1	2	ND	ND	ND	0
		PV3	4	ND	ND	ND	2
	Venegiota	V2	4	ND	ND	ND	0
		V4	6	ND	ND	ND	0

*Note:* Adults: > 3 years; Subadults (2–3 years); Juveniles (1–2 years), Pups (< 1 year).

In the family units belonging to Busabella and Fosse, the number of individuals was well known, as all the individuals born in the previous years were captured and marked with ear tags, since the areas were included in a long‐term study (Forti et al. 2025). In Val Venegia, the number of individuals was estimated through periodic censuses using the method of observation from vantage points (Forti et al. 2025).

From mid‐June to late September, the study area is utilized for seasonal grazing by domestic livestock, primarily cattle herds (about 60 dairy cows in Busabella and Fosse and about 200 heads, including cows and heifers in Venegiota e Piani della Vezzana), with occasional presence of sheep flocks. This anthropogenic influence on the ecosystem is complemented by the year‐round presence of several native ungulate species, which include Red deer ( *Cervus elaphus* L.), Roe deer ( *Capreolus capreolus* L.), and Northern chamois ( *Rupicapra rupicapra* L.).

The study area consistently harbors both natural potential predators of the marmot (Forti et al. 2025), including Red fox ( *Vulpes vulpes* L.), Wolf ( *Canis lupus* L.), and raptors such as Golden eagle ( *Aquila chrysaetos* L.), and Eurasian goshawk (*Astur gentilis* L.), as well as human‐associated predators like shepherd dogs and dogs accompanying tourists. In recent years, predator–prey interactions have been observed between the marmot and all the mentioned species. Although canine predation cannot be entirely ruled out, it is considered less likely to occur. This reduced probability is due to the absence of feral or stray dogs in the area, and the fact that shepherd dogs and tourist‐owned dogs are typically kept under human supervision, primarily out of concern for potential wolf predation on dogs themselves.

### 2.2 Study Design and Sampling

To elucidate the dietary composition and foraging strategies of alpine marmots ( *Marmota marmota* L.) across diverse alpine pasture ecosystems throughout the summer season, a comprehensive study encompassing multiple analytical approaches was conducted. Although it is a well‐documented phenomenon that marmots, particularly upon emergence from hibernation, may incorporate foods of animal origin, notably insects, into their dietary regimen (Bassano et al. 1996; Garin et al. 2008), the present study focused exclusively on the vegetative component of their diet since it is more directly related to pasture management.

Data collection spanned from early June to late August 2022, with sampling regularly covering all 9 family units every month. The methodological approach integrated three primary components that are thoroughly described in the following paragraphs:

1. Floristic composition analysis: A systematic survey of plant species present in the pastures was conducted to establish baseline vegetation profiles for each study area.
2. Herbage nutritional analysis: Representative herbage samples (*n* = 450) were collected and subjected to comprehensive nutritional composition analysis to assess the qualitative aspects of available forage.
3. Marmot fecal pellet analysis: DNA metabarcoding techniques were employed to analyze marmot fecal pellets (*n* = 50), enabling high‐resolution dietary reconstruction.

#### 2.2.1 Pasture Floristic Analysis, Plant Sampling, and Fecal Collection Methodology

For each marmot family unit, the main burrow was identified, which corresponded to the hibernaculum—the winter refuge typically situated at the approximate centre of the family's home range (Perrin et al. 1993). These main burrows were characterized by distinctive features such as earth mounds, multiple entrances, and high utilization rates (Ballová and Šibík 2015). To conduct comprehensive botanical surveys and analyze herbage composition, a circular sampling area with a 100‐m radius was delineated and centered on each main burrow. This methodological approach was informed by previous research indicating that primary burrows serve as focal points for summer activity (Perrin et al. 1993; Ballová and Šibík 2015; Wang and Hou 2021), and that average family home ranges (HR) rarely exceed a 100‐m radius (3.14 ha) (Forti et al. 2025).

Within each family unit's sampling area, five 10‐m linear transects were established, positioned to represent the available vegetation types. An exception was made for the PV area, where, because of homogeneous vegetation, only four and three transects were established for family units PV1 and PV3, respectively, resulting in a total of 42 sampling transects across all study sites.

**FIGURE 3:**
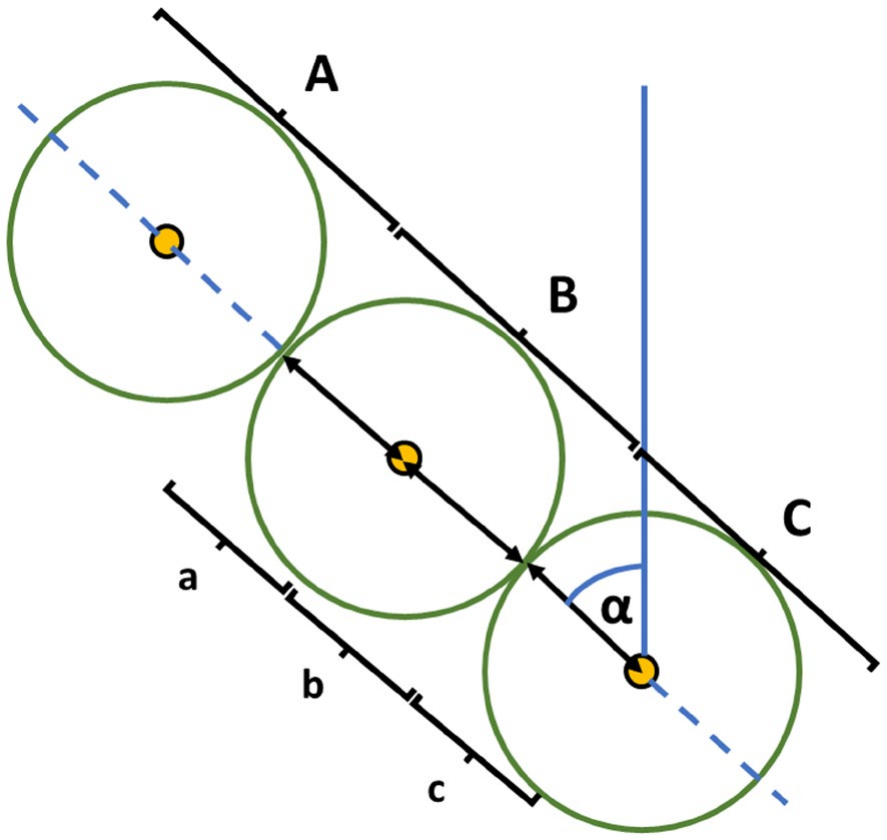
Linear transect (10 m, a + b + c) used to perform floristic analysis (Daget‐Poissonet point‐intercept method) and circular plots (A, B, C) used to calculate dung pats density, collect herbage samples (1 sample for each segment a, b and c) and estimate grass length along the linear transect. Α represents the 45° angle of the linear transect relative to the maximum slope gradient.

Linear transects were oriented at a 45° angle relative to the maximum slope gradient (Figure 3) and were demarcated using 15 pins placed at 70 cm intervals along a string. The Daget‐Poissonet point‐intercept method (Daget and Poissonet 1971) was employed for botanical surveys. Species identification adhered to the Flora d'Italia (Pignatti et al. 2017) taxonomic guide. For each transect, the relative abundance of each plant species was calculated to determine the proportional composition of the pasture, following the Daget and Poissonet equation (Daget and Poissonet 1971). These transects also served as reference points for estimating cattle fecal density, assessing grass length, and collecting herbage samples for nutritional composition analysis. To facilitate detailed sampling, each transect was subdivided into three equal segments (3.30 m each), labeled a, b, and c. Additionally, three distinct circular areas (radius 3.30 m) were established along the transect, designated as A, B, and C, as illustrated in Figure 3. This rigorous sampling design enabled a comprehensive assessment of the floristic composition, vegetation structure, and associated ecological parameters within the marmots' habitat, providing a robust foundation for subsequent dietary analyses.

Within each circular zone (A, B, and C) along the transects, cattle dung density was quantified by enumerating dung pats with a diameter ≥ 20 cm. The mean dung pat density per transect was calculated by averaging the density values from each circular area. Vegetation height was assessed using a stratified sampling method. Within each circular zone, grass length at eight representative locations, chosen to proportionally reflect the spatial distribution of height variations, was measured. These measurements were then averaged to obtain a mean grass height for each transect.

Herbage samples were collected along each transect (Figure 3), approximately 1 m adjacent to segments a, b, and c. Vegetation was harvested using a brush cutter, cutting approximately 2 cm above the ground level.

Marmot fecal pellets were collected on a biweekly basis from early June through late August and then assigned to each month. Fresh droppings were obtained from known latrines, where available, or near the entrances of main burrows for each family unit. Samples were promptly refrigerated and then stored at −20°C pending DNA analysis.

#### 2.2.2 Laboratory Analyses

Herbage samples after being dried at 60°C for 24 h and ground at an average diameter of 1 mm were analyzed for dry matter (DM), ash, crude protein (CP), ether extract (EE), neutral detergent fiber (NDF), following the procedures reported in literature (Association of Official Analytical Chemists 2003; ANKOM Technology 2020). Non‐fiber carbohydrates (NFC) were calculated through the following formula: NFC = 100—Ash—CP—EE—NDF, where all the variables are expressed as the DM basis (Association of Official Analytical Chemists 2003). The procedure for the determination of herbage FA composition started with a Soxhlet extraction through the SoxROC Extraction Unit (OPSIS LiquidLINE, OPSIS AB, Furulund, Sweden) utilizing petroleum ether with a boiling range of 40°–60°C as solvent, according to the AOAC Method 2003.05 (Association of Official Analytical Chemists 2016). It is important to note that during this extraction, the sample was not dried to completion, preserving the integrity of the extracted compounds. Following extraction, the sample underwent a trans‐esterification reaction. The trans‐esterification was carried out using a 4% methanolic sulfuric acid solution. The reaction mixture was incubated overnight in an oven maintained at 60°C, ensuring complete conversion of the fatty acids. After the trans‐esterification process, the resulting fatty acid methyl esters (FAMEs) were suspended in n‐heptane, preparing them for chromatographic analysis. The chromatographic separation was achieved employing Gas Chromatography with Flame Ionization Detection (GC‐FID), using a Shimadzu system (GC‐2010 Plus) equipped with an Omegawax (Sigma‐Aldrich Co. LLC., Saint Louis, USA) 250 column (30 m × 0.25 μm × 0.25 μm), according the method described by Kokošková et al. (2025). Obtained data were reported as percentage of the total detected fatty acids.

Marmots' fecal samples were analyzed for the presence of plant specimens through metabarcoding analysis. Total DNA was extracted using the Mag‐Bind stool DNA 96 kit (Omega Bio‐tek, GA, USA) according to the manufacturer's instructions. Plant species identification was performed by sequencing the chloroplast trnL (UAA) intron region using the primers A49325: 5′ CGAAATCGGTAGACGCTACG3 3′ and B49466: 5′ CCATTGAGTCTCTGCACCTATC 3′ (Taberlet et al. 2007) as reported in Palumbo et al. (2021) with the following cycling conditions: initial denaturation step at 95°C for 2 min; 35 cycles at 95°C for 15 s, 52°C for 15 s, 72°C for 30 s; final extension step at 72°C for 5 min.

Raw reads were processed with MICCA (v.1.7.2) software (Albanese et al. 2015). Specifically, the paired end reads were merged using VSEARCH (https://github.com/torognes/vsearch), with a minimum overlap length of 100 and a maximum number of allowed mismatches of 32. Primers were trimmed using Cutadapt v.1.18 (Martin 2011), and merged reads shorter than 100 bp with an expected error rate higher than 0.25% were removed. Filtered sequences were clustered into Amplicon Sequence Variants (ASV) using the UNOISE (https://doi.org/10.1101/081257) algorithm available in MICCA. Taxonomic assignment was carried out using the RDP classifier v. 2.13 (Wang et al. 2007) against the BioAir reference database (Leontidou et al. 2018) for trnL.

#### 2.2.3 Sequence Analysis and Taxonomic Assignment

Following initial sequence processing, a comparative analysis of unidentified sequences using the BLAST (Basic Local Alignment Search Tool) algorithm was conducted against the NCBI nucleotide database (https://blast.ncbi.nlm.nih.gov/Blast.cgi?PROGRAM=blastn&PAGE_TYPE=BlastSearch&LINK_LOC=blasthome) to enhance taxonomic resolution. For each fecal sample, the occurrence of sequences corresponding to distinct taxa was quantified. To facilitate inter‐sample comparisons, these counts were normalized to proportions, henceforth referred to as “diet proportions”.

The interpretation of DNA metabarcoding data is susceptible to potential technical biases (Pompanon et al. 2012; Ando et al. 2018; Littleford‐Colquhoun et al. 2022; Stapleton et al. 2022). However, previous comparative studies with traditional micro‐histological methods have demonstrated that DNA metabarcoding facilitates improved sensitivity and accuracy in detecting species present in the diets of wild herbivores, notably wild rodents (Soininen et al. 2009). Nevertheless, since the quantitative information obtained through metabarcoding, on the basis of relative read abundance (RRA), can have certain limitations because of potential primer mismatches and variations in the correlation between abundance in feces and true dietary content among different species (Stapleton et al. 2022; Sato 2024), we utilized both RRA and relative frequency of occurrence (FOO) to quantify dietary data, as reported by Ando et al. (2018).

To address discrepancies between our reference libraries and the local flora, a rigorous taxonomic reconciliation process was implemented. This involved cross‐referencing our sequence assignments with known taxa distributions in Northern Italy, particularly Trentino, and with our data from floristic analysis. Taxa absent from the region were reassigned to higher taxonomic levels (e.g., Genus A species B to Genus A sp.). Conversely, when a genus or family was represented by a single species or genus in the region, assignments were refined accordingly (e.g., *Bistorta* sp. to *Bistorta vivipara* , Celastraceae to *Parnassia* sp.). In cases where sequences were initially assigned to species not present in the region, they were grouped with congeneric species known to occur locally (e.g., various *Vaccinium* species consolidated under *Vaccinium* spp.). This comprehensive approach to sequence analysis and taxonomic assignment ensures a robust and ecologically relevant interpretation of the metabarcoding data, tailored to the specific floristic composition of the study area.

### 2.3 Calculations and Statistical Analysis

#### 2.3.1 Analytical Framework for Plant Abundance and Dietary Selection

The MicrobiomeAnalyst platform was employed, whose tools are suited to handle datasets of any kind (https://www.microbiomeanalyst.ca/MicrobiomeAnalyst/) to process and compare plant abundance data at family, genus, and species levels for both pasture composition and fecal samples. Pasture data were derived from the Daget‐Poissonet point‐intercept method, whereas fecal sample data were obtained through metabarcoding analysis. Data were imported as OTU abundance tables, accompanied by taxonomic and metadata information.

#### 2.3.2 Data Filtration and Normalization

Owing to potential contamination (Ando et al. 2018), polymerase chain reaction and sequencing errors, tag jumps, and biological and technical biases to generate low‐quality and erroneous sequences, OTUs that had an identical value across all samples, and those found just in a solitary fecal sample were ignored. The same procedure was applied to pasture data. Rarefaction curves were generated for the pasture data from each study area. Regarding fecal samples, although a seemingly straightforward solution is to discard low‐abundance sequences to mitigate errors, doing so by setting an arbitrary threshold without considering data patterns can erroneously eliminate genuine dietary sequences in a manner that impacts downstream inferences (Littleford‐Colquhoun et al. 2022). Consequently, instead of filtering abundance data using arbitrary thresholds, Hill numbers on the basis of both abundance and incidence were calculated, in accordance with the methodology proposed by Alberdi and Gilbert (2019). Hill numbers constitute a mathematically unified family of diversity indices that quantify diversity in terms of the effective number of species (Hill 1973), thereby enabling researchers to assign greater or lesser weight to rare taxa by modulating the scaling parameter q (Alberdi and Gilbert 2019).

Hill numbers, denoted as ^q^D, represent the effective number of species in a community, where *q* is the order of diversity that determines the sensitivity to species relative abundances. Given D as this diversity:

1. *q* = 0 (Species Richness): ^0^D = S. Quantifies the richness encompassing all species, where *S* is the total number of species in the community.
2. *q* ≈ 1 (Shannon Diversity): ^1^D = exp.(H′). Quantifies the diversity of “typical” species, where H′ = −Σ(p_i × ln(p_i)), and p_i is the proportion of species *i* in the community. This is equivalent to the exponential of Shannon entropy. We used *q* = 0.9999 in calculations because of mathematical constraints.
3. *q* = 2 (Simpson Diversity): ^2^D = 1/λ. Quantifies the diversity of dominant species, where *λ* = Σ(p_i^2^), and p_i is the proportion of species i in the community. This equals the inverse of Simpson's concentration index.

The general formula for Hill numbers is: ^q^D = (Σp_i^q^)^(1/(1−q))^.

As q increases, the measure becomes less sensitive to rare species and more influenced by common species (Chao et al. 2014; Littleford‐Colquhoun et al. 2022).

Hill number profiles were constructed on the basis of both incidence and abundance data for diets across different macro‐areas and months (Alberdi and Gilbert 2019). For each macro‐area, ranked lists of the most important species were compiled, considering two diversity orders: *q* ≈ 1 (representing typical species) and *q* = 2 (representing dominant species). The importance of species was calculated differently for each q value:

- For *q* ≈ 1, species importance equates to its proportional abundance in the community (p_i).
- For *q* = 2, species importance is calculated as: Importance(i,j) = (p_ij)^2^/Σ(p_ij)^2^, where p_ij is the relative abundance of species *i* in sample *j*.

This approach allowed us to assess species importance from both a balanced (*q* ≈ 1) and a dominance‐focused (*q* = 2) perspective (Chao et al. 2014).

All subsequent comparisons between diets across areas and months, as well as the calculation of plant selection indices (see the following paragraphs) and the correlation between plant species in the diet and pasture characteristics, were conducted using the list of species selected on the basis of the incidence‐based Hill number of the order of diversity *q* ≈ 1, indicating typical species (Littleford‐Colquhoun et al. 2022). All datasets were subsequently normalized using Total Sum Scaling (TSS).

#### 2.3.3 Plant Selection Analysis

Despite acknowledging the many biological and methodological factors that potentially skew the representation of species in fecal dietary samples (Sato 2024), it was sought to elucidate whether the consumption of plant species correlated with their availability in the grazing environment. To this end, the abundance of sequence reads was employed as a proxy for estimating the relative biomass contribution of various species to the diet composition. Recent research by Stapleton et al. (2022), in fact, has provided empirical evidence supporting a positive, albeit moderate, quantitative relationship between sequence read abundance and the actual quantities of ingested plant material. To see whether or not plant ingestion by marmots followed plant availability in the pasture, for each area, the average Jacobs's selection index at the genus level (Jacobs 1974) was calculated following the formula:

D = (*r*—*p*)/(*r* + *p*—2*rp*), where *r* represents the relative abundance of a resource in the diet, and *p* denotes its relative availability in the environment. This index ranges from −1 to +1, with negative values indicating avoidance, positive values suggesting preference, and values approximating zero implying that the resource is utilized in proportion to its environmental abundance. In the calculation of D, for each area, only genera that, in the pasture and/or in the diet accounted for at least 2%, were included.

#### 2.3.4 Statistical Analyses

1. Reingold–Tilford Heat Tree Analysis (Reingold and Tilford 1981): This method was utilized to assess differences in plant species proportions in diet (abundance) across different months. This approach leverages taxonomic hierarchies to quantitatively and statistically depict community differences using median abundance and the Wilcoxon Rank Sum test (*p* < 0.05).
2. Linear Discriminant Analysis Effect Size (LEfSe): This technique was employed to identify species with significant differential abundance between areas or months. The *p*‐value cut‐off was set to 0.05 and the Log LDA score cut‐off to 3.0. LEfSe performs the Kruskal–Wallis test to identify features (e.g., botanical species) with significantly different values among the sample classes. For these features, LEfSe then applies linear discriminant analysis (LDA) to estimate the effect size relevance of each feature. The Log LDA score is the logarithm of the effect size estimated by the LDA analysis. Higher Log LDA scores indicate more powerful features in discriminating the sample classes (Segata et al. 2011).
3. Correlation Analysis: To elucidate drivers of marmot dietary choices, correlations between diet composition and pasture characteristics were investigated. Pasture variables included nutritional composition (dry matter—DM, crude protein—CP, ether extract—EE, neutral detergent fiber—NDF, non‐fiber carbohydrates—NFC), fatty acid profiles (saturated fatty acids (SFA), monounsaturated FA (MUFA), polyunsaturated FA (PUFA), Omega‐6 FA (n6), Omega‐3 FA (n3)), grass length, and bovine dung pat density. These variables were discretized into five categories (from 1 to 5) before Spearman correlation analysis.
4. Preference Index: In evaluating pasture quality, particularly for non‐ruminant species, high DM and NDF content are generally considered indicators of lower nutritional value (Wasserman and Chapman 2003; White 2008). Conversely, elevated levels of CP, EE, representing fat content, and NFC, primarily sugars, are associated with higher quality forage (Wasserman and Chapman 2003; White 2008; Aryal et al. 2015). To elucidate the relationship between plant species consumption and pasture nutritional quality, we developed a novel preference index. This index aims to identify species that are preferentially consumed in high‐quality pastures (potentially contributing to the overall nutritional value) and those consumed in low‐quality pastures (potentially possessing above‐average nutritional value relative to the surrounding vegetation). Plant species exhibiting significant correlations (*p* < 0.05) with at least one of the key nutritional variables (DM, CP, EE, NDF, and NFC) were selected. For each significant correlation, a value on the basis of the following criteria was assigned:
  - Positive value, equal to the absolute value of the correlation coefficient, was assigned for:
    ○ Positive correlations with CP, EE, and NFC.
    ○ Negative correlations with DM and NDF.
  - Negative value of the correlation coefficient was assigned for:
    ○ Negative correlations with CP, EE, and NFC.
    ○ Positive correlations with DM and NDF.

This scoring system allows for a nuanced interpretation of species preferences in relation to pasture nutritional composition. Species with higher positive scores are likely to be associated with higher quality pastures, whereas those with negative scores may indicate consumption patterns linked to lower quality foraging areas.

This preference index methodology was extended to incorporate additional ecological variables, aiming to elucidate the relationship between plant species consumption and broader environmental factors. This expanded analysis encompassed the following parameters:

1. *Vegetation structure*: The correlation between plant species consumption and grass height was assessed, assigning values as follows:
  ○ Short grass: negative correlation coefficient
  ○ Tall grass: positive correlation coefficient
2. *Cattle presence*: The density of cattle dung pats (measured as the number of dung pats per m^2^) was used as a proxy for bovine presence in the area. Correlations were scored as:
  ○ Low dung pat density: negative correlation coefficient.
  ○ High dung pat density: positive correlation coefficient
3. *Fatty acid composition*: The relationship between plant species consumption and the fatty acid profile of the pasture was evaluated, considering the following categories:
  ○ Saturated Fatty Acids (SFA).
  ○ Monounsaturated Fatty Acids (MUFA).
  ○ Polyunsaturated Fatty Acids (PUFA).
  ○ Omega‐6 Fatty Acids (n6)
  ○ Omega‐3 Fatty Acids (n3)

For each of these fatty acid categories, correlations were scored using the same system on the basis of the direction of the relationship.

This approach provides a quantitative framework for assessing the interplay between plant species selection and pasture nutritional dynamics, offering insights into the foraging strategies of alpine marmots in heterogeneous nutritional landscapes.

## 3 Results

In the pastures across the entire study area, 158 plant species belonging to 36 families were identified, of which 11 accounted for at least 1% abundance. Overall, only 114 species accounted for at least 0.02% in the pasture (Table S1 and Figure S2) and were considered for the evaluation of plant species availability in the pasture. The rarefaction curves (Figure S1) demonstrate asymptotic behavior, indicating that our sampling effort has captured a significant portion of the plant species diversity in each area. Regarding the alpine marmot's diet, 50 fecal samples were collected from four different locations: Busabella (12), Fosse (12), Piani della Vezzana (10), and Venegiota (16). Samples were successfully obtained from all areas throughout the study period, although June was overrepresented with 23 samples, followed by July (16) and August (11). Upon the plant identification through fecal metabarcoding, overall, 119 species (Table S2) belonging to 48 families were found, of which 14 accounted for at least 1% abundance. Not‐assigned species accounted for 1.64% abundance.

**FIGURE 4:**
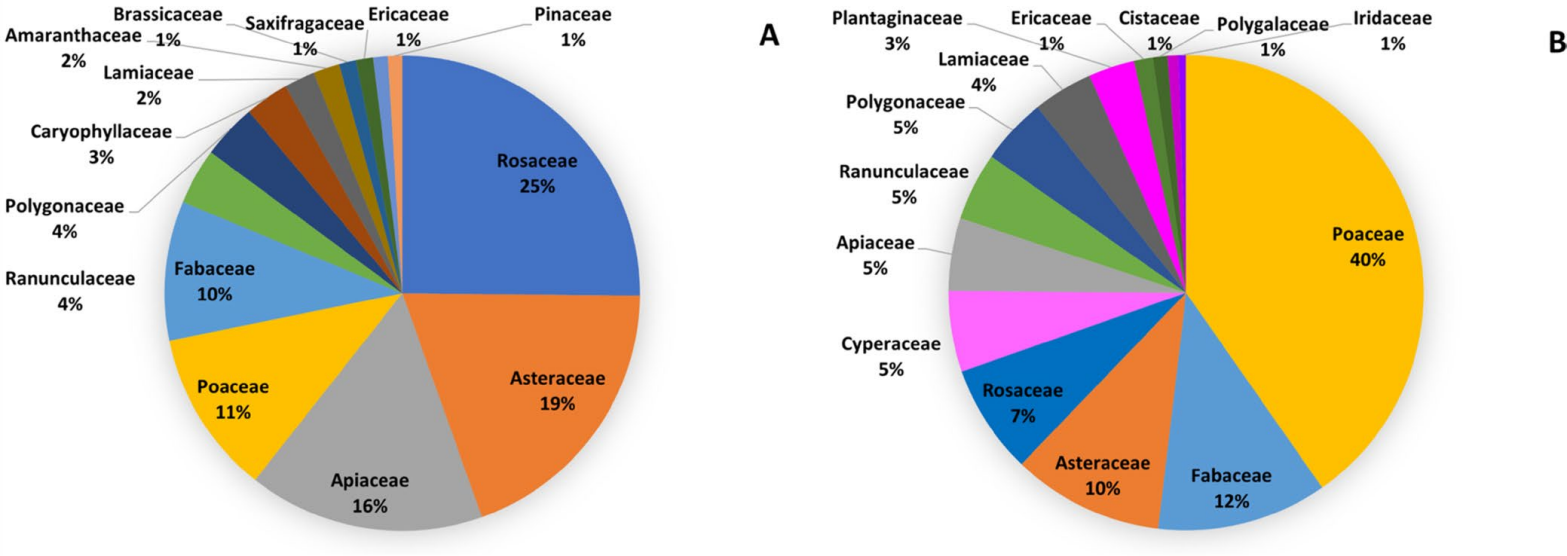
Overall abundance (%) of the 14 main plant families (at least 1% abundance) found in alpine marmot fecal pellets (A) and pastures (B). Families present in both A and B are represented by the same color.

Figure 4 illustrates the overall proportion of the 14 main plant families found in both feces (Figure 3A) and pastures (Figure 3B). The proportions of the shared families differed; for example, Poaceae, the most abundant family in the pastures (40%), ranked only fourth in the feces (11%), whereas Rosaceae accounts for 7% of pasture abundance and 25% in feces. The complete lists of families and species found in pastures and feces are reported in Tables S1 and S2, respectively. Overall, some species or genera were present in all (*Alchemilla vulgaris, Centaurea* spp., *Taraxacum officinale*) or almost all of the fecal samples collected, such as *Carum carvi*, *Leontodon hispidus* , and *Petasites albus*, among others (Table S2). Other species, such as *Schistidium* spp. and *Setaria verticillata* , were found very rarely, appearing only twice out of 50 samples.

**FIGURE 5:**
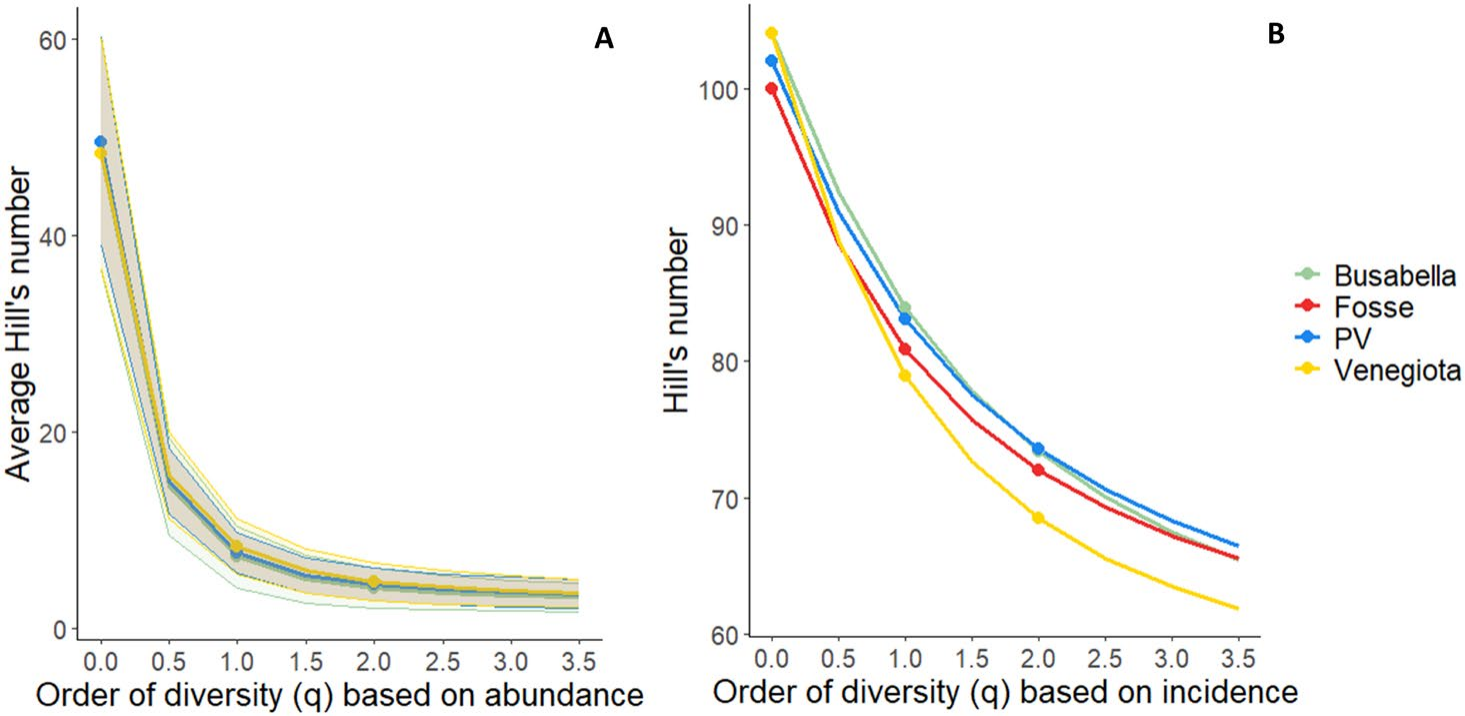
(A) Average Hill numbers profile related to species abundance in fecal samples at varying q values in different areas. Fosse lines are not visible because of overlapping. Thick lines represent the average, whereas light colors represent the standard deviation. (B) Hill numbers profile related to species incidence in fecal samples at varying q values in different areas, where *q* = 0 is the richness, *q* = 1 represents typical plant species, and *q* = 2 represents the dominant species in the diet.

The average fecal sample richness (for *q* = 0; ^0^D = S) for each area ranged from 48.3 species in Venegiota to 50 species in PV (Figure 5A), with the maximum (72) reached in a sample from Venegiota in June and the minimum (26) found in a sample from Busabella in August. The overall richness (among all samples) in the areas was highest in Venegiota and Busabella (106), followed by Piani della Vezzana (104) and Fosse (102). Considering values of *q* ≈ 1 (Shannon Diversity) and *q* = 2 (Simpson Diversity) on the basis of the abundance, the Hill numbers of typical species and dominant species dropped on average to a range of (6–10) to (4–7), respectively, with the highest values in Piani della Vezzana, as reported in Table 2.

**TABLE 2:** List of typical (*q* ≈ 1) and dominant (*q* = 2) species in the marmot diet in different macro‐areas according to abundance‐based Hill numbers and species relative importance (Imp.), frequency (Freq.), and mean abundance.

Macroarea	Typical Species (q ≈ 1)				Dominant species (q = 2)			
	Species	Import.	Freq.	Mean abundance	Species	Import.	Freq.	Mean abundance
Busabella	*Alchemilla vulgaris*	0.252	1.000	0.252	*Alchemilla vulgaris*	0.341	1.000	0.252
	*Stellaria nemorum*	0.076	0.750	0.076	*Stellaria nemorum*	0.131	0.750	0.076
	*Taraxacum officinale*	0.065	1.000	0.065	*Trifolium pratense*	0.082	0.917	0.063
	*Trifolium pratense*	0.063	0.917	0.063	*Rhodiola rosea*	0.080	0.167	0.058
	*Festuca rubra*	0.059	1.000	0.059				
	*Rhodiola rosea*	0.058	0.167	0.058				
	*Helianthemum* spp.	0.056	0.750	0.056				
	***Total abundance***			**0.629**				**0.448**
Fosse	*Carum carvi*	0.145	1.000	0.145	*Carum carvi*	0.197	1.000	0.145
	*Helianthemum* spp.	0.105	1.000	0.105	*Onobrychis viciifolia*	0.151	0.583	0.101
	*Onobrychis viciifolia*	0.101	0.583	0.101	*Alchemilla vulgaris*	0.128	1.000	0.085
	*Alchemilla vulgaris*	0.085	1.000	0.085	*Helianthemum* spp.	0.111	1.000	0.105
	*Taraxacum officinale*	0.059	1.000	0.059				
	*Achillea millefolium*	0.058	1.000	0.058				
	*Poa alpina*	0.057	1.000	0.057				
	*Trifolium pratense*	0.037	0.833	0.037				
	***Total abundance***			**0.647**				**0.437**
Piani della Vezzana	*Alchemilla vulgaris*	0.101	1.000	0.101	*Alchemilla vulgaris*	0.166	1.000	0.101
	*Helianthemum* spp.	0.076	1.000	0.076	*Helianthemum* spp.	0.115	1.000	0.076
	*Poa alpina*	0.064	1.000	0.064	*Biscutella leavigata*	0.077	0.800	0.053
	*Prunella vulgaris*	0.055	0.900	0.055	*Prunella vulgaris*	0.072	0.900	0.055
	*Biscutella leavigata*	0.053	0.800	0.053	*Trifolium pratense*	0.070	0.900	0.044
	*Leontodon hispidus*	0.052	0.900	0.052	*Poa alpina*	0.066	1.000	0.064
	*Crepis* spp.	0.048	1.000	0.048	*Ranunculus acris*	0.050	0.900	0.043
	*Phleum pratense*	0.044	0.800	0.044				
	*Trifolium pratense*	0.044	0.900	0.044				
	*Ranunculus acris*	0.043	0.900	0.043				
	*Lotus corniculatus*	0.041	0.900	0.041				
	***Total abundance***			**0.621**				**0.435**
Venegiota	*Alchemilla vulgaris*	0.299	1.000	0.299	*Alchemilla vulgaris*	0.458	1.000	0.299
	*Carum carvi*	0.183	1.000	0.183	*Carum carvi*	0.281	1.000	0.183
	*Taraxacum officinale*	0.074	1.000	0.074	*Taraxacum officinale*	0.064	1.000	0.074
	*Trifolium repens*	0.032	0.813	0.032	*Picea abies*	0.038	0.563	0.022
	*Festuca rubra*	0.032	1.000	0.032	*Chenopodium* spp.	0.033	0.563	0.023
	*Centaurea* spp.	0.031	1.000	0.031				
	*Ranunculus acris*	0.027	1.000	0.027				
	*Achillea millefolium*	0.026	0.875	0.026				
	***Total abundance***			**0.703**				**0.601**

*Note:* Total abundance (from 0 to 1) is calculated as the sum of relative abundance of typical and dominant species in fecal samples belonging to each macro‐area. The bold values represent the sum of the mean abundance of the species selected by the Hill numbers based on abundance for *q* = 1.

As reported in Figure 5B the Hill number profiles for each macro‐area on the basis of incidence (frequency) maintain higher values for typical and dominant species, which range from 79 (Venegiota) to 84 (Busabella) and from 68 (Venegiota) to 76 (Busabella), respectively.

**FIGURE 6:**
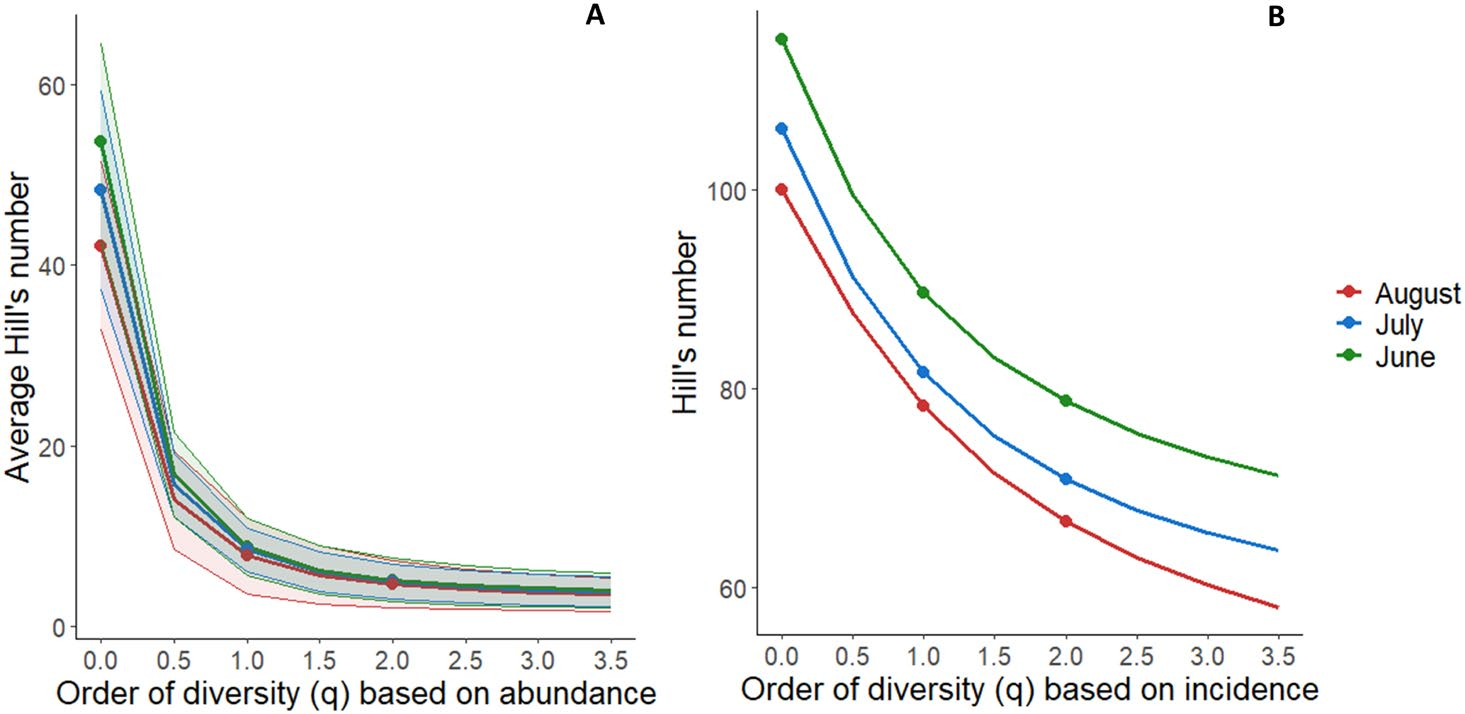
Hill numbers profile related to species abundance (A) and incidence (B) in fecal samples at varying *q* values in different months, where *q* = 0 is the richness, *q* = 1 represents typical plant species, and *q* = 2 represents the dominant species in the diet. Thick lines represent the average, whereas light colors represent the standard deviation.

Overall, richness in the marmot diet changed over the summer, being highest in June and lowest in August. The same trend is visible for Shannon diversity (*q* ≈ 1) and Simpson diversity (*q* = 2) when based on incidence, whereas they are similar when based on abundance, as reported in Figure 6.

The stacked bar graphs depicting species abundance in pastures and marmot feces per macro‐area are shown in Figures S2 and S3, respectively. As regards fecal samples, we considered 86 (overall *q* ≈ 1 on the basis of incidence) species to relate the marmot diet to plants' availability in pastures and other factors.

**FIGURE 7:**
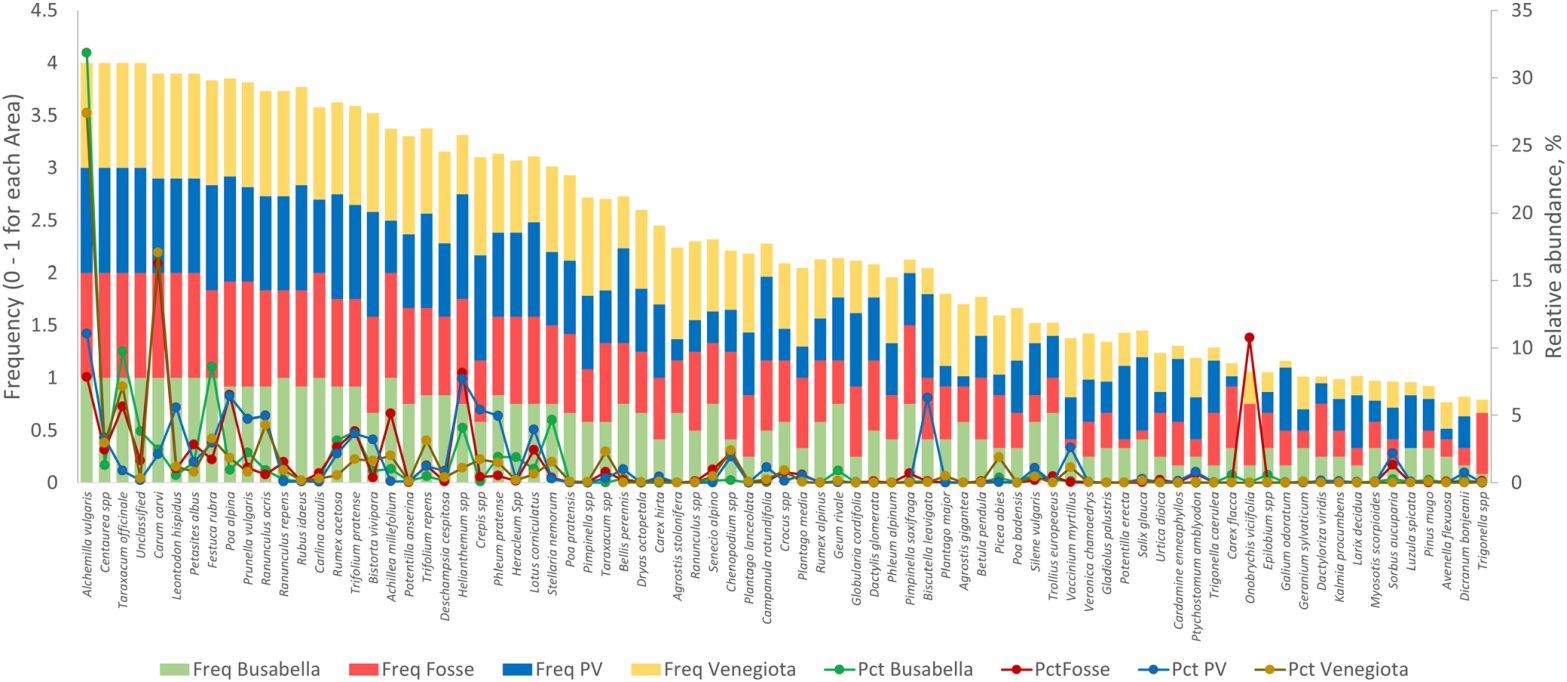
List of plant species found in diets with a proportion of at least 0.01 (1%) and their overall frequency. PV = Piani della Vezzana.

Figure 7 summarizes the frequency and the abundance of the main species (abundance at least 1%) in the diet of marmots belonging to different macro‐areas. 22 species showed an average abundance of at least 5% in at least one macro‐area. *Alchemilla vulgaris* showed the highest average abundance with peaks of 32% and 27% in Busabella and Venegiota, respectively, followed by *Carum carvi* (17%) in Venegiota and *Onobrychis viciifolia* (10.7%) in Fosse.

**FIGURE 8:**
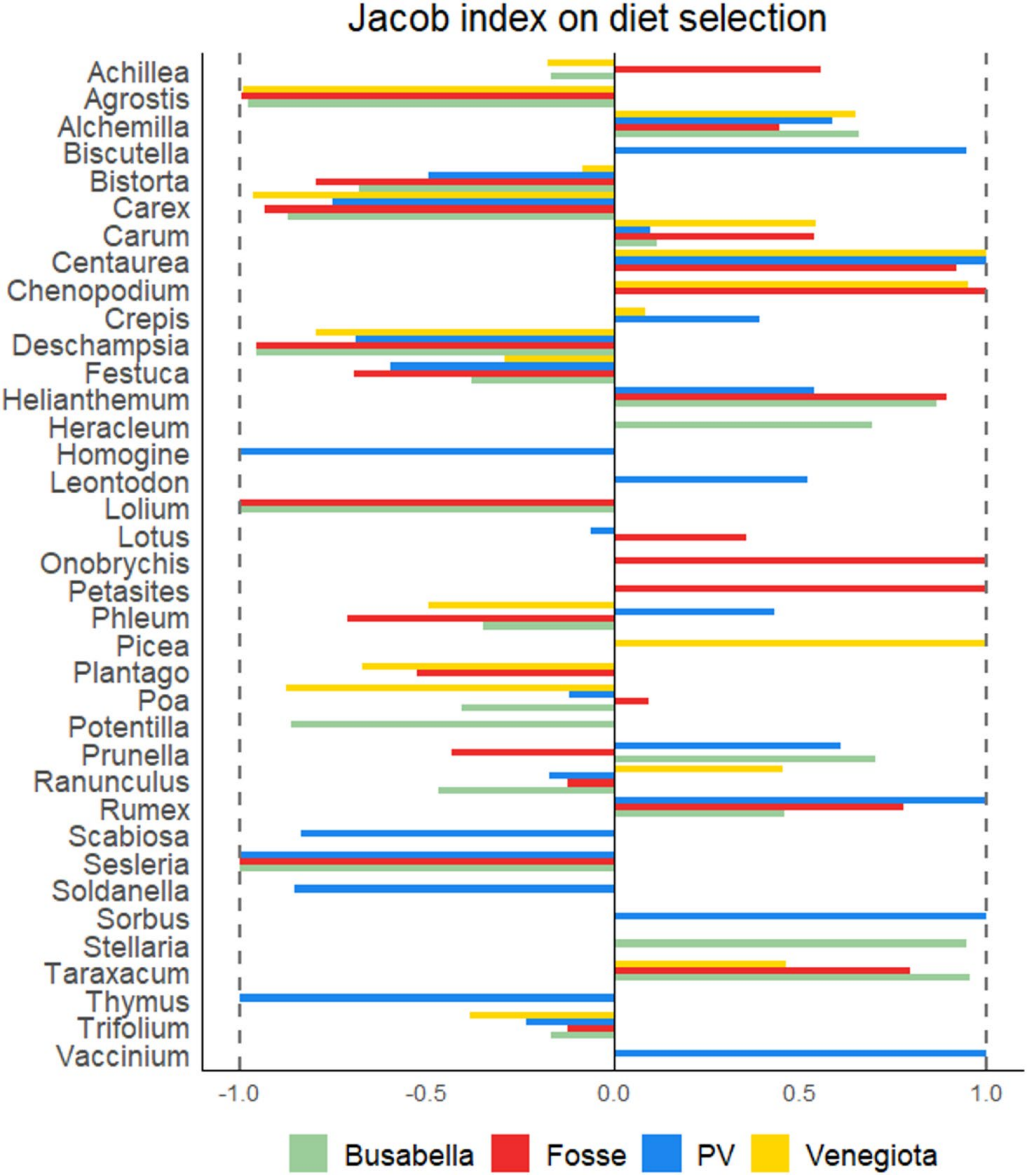
Jacob's index of plant genera accounting in pasture or in diet for at least 2% within each area. PV = Piani della Vezzana.

As reported in Figure 8, out of 37 genera included in the calculation of the Jacob's index, 23 were positively selected in at least one area. *Alchemilla and Carum* were positively selected in all the areas, whereas *Bistorta*, *Carex*, *Deschampsia*, *Festuca*, and *Trifolium* were consumed below their availability in all the areas. *Achillea*, *Lotus*, *Phleum*, *Poa*, *Prunella*, and *Ranunculus* were negatively or positively selected according to the area considered. Among the 14 genera, which were positively selected in one single area, *Heracleum* and *Stellaria* were selected in Busabella, *Achillea*, *Lotus*, *Onobrychis*, *Petasites*, and *Poa* in Fosse, *Biscutella*, *Leontodon*, *Phleum*, *Sorbus*, and *Vaccinium* in Piani della Vezzana, and *Picea* and *Ranunculus* in Venegiota.

**FIGURE 9:**
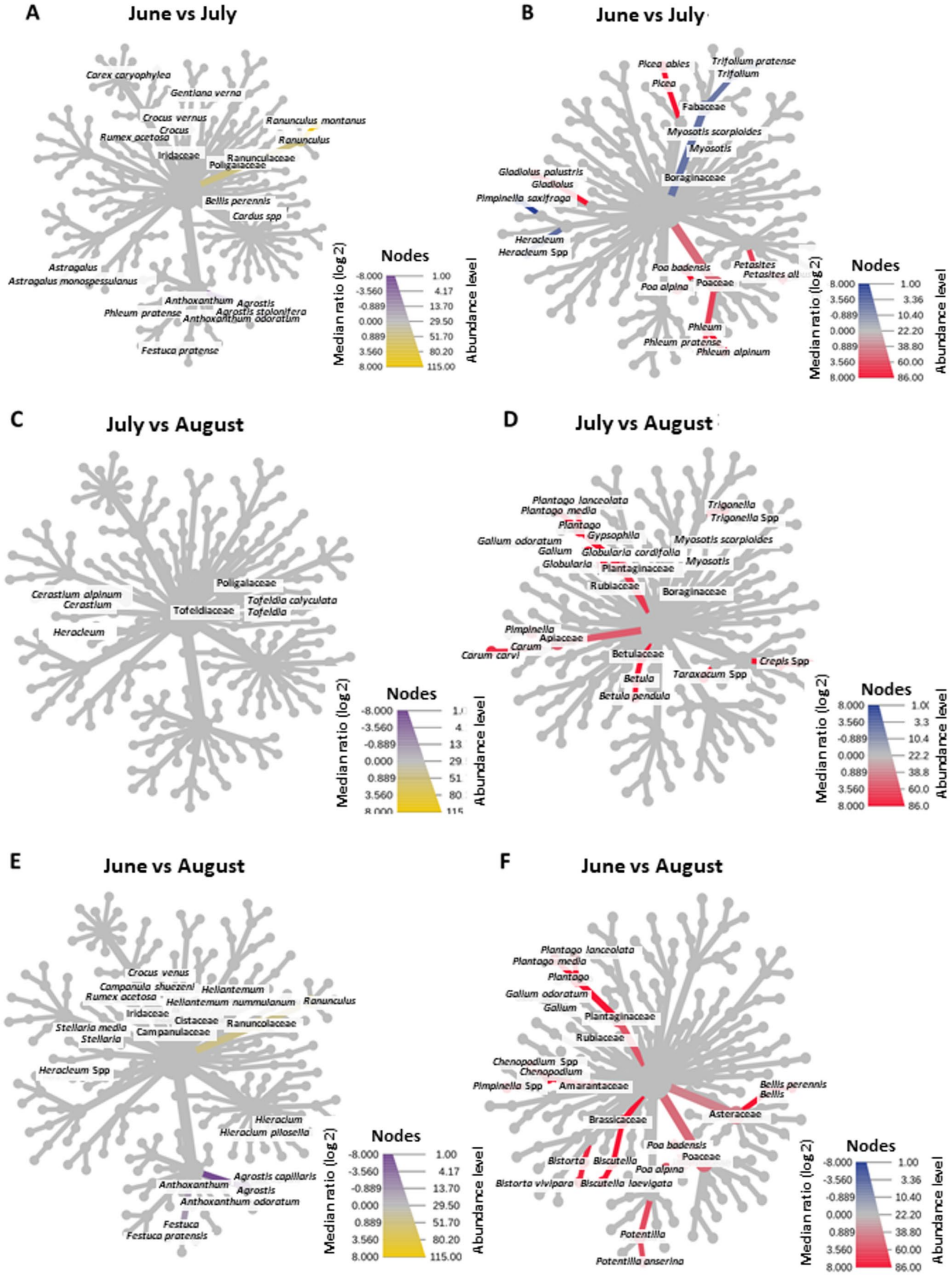
Reingold–Tilford Heat Trees representing comparisons of plant abundance among different months (June, July, and August) in pastures (A, C, and E) and in marmot fecal samples (B, D, and F). Colored branches (not gray) indicate a significant statistical difference (*p* < 0.05).

To determine the extent to which the marmot's diet differed over the summer and whether this difference was due solely to plant proportions, direct comparisons among 3 months (June, July, and August) were conducted. The results of these comparisons are represented as Reingold–Tilford Heat Trees (Reingold and Tilford 1981), as shown in Figure 9.

The variability in species availability among seasons was very low (Figure 8A,C,E), with significant differences found only in June, where Ranunculaceae were higher and *Agrostis* spp. and *Festuca* spp. were lower.

Diet, however, changed (Figure 8B,D,F). The June diet is characterized by higher content of Poaceae (*Phleum* spp., *Poa* spp.), Asteraceae (*Petasites albus*), Iridaceae (*Gladiolus palustris*), and Pinaceae ( *Picea abies* ), whereas compared to July, the June diet is poorer in Fabaceae ( *Trifolium pratense* ), *Pimpinella saxifraga* , and *Heracleum* spp. Between July and August, the July diet is higher in Plantaginaceae (*Globularia cordifolia*, *Plantago* spp.), Apiaceae (*Carum carvi, Pimpinella* spp.), Betulaceae ( *Betula pendula* ), *Crepis* spp., *Taxacacum* spp., and *Trigonella* spp. The greatest difference in diets is found between June and August, where the June diet is richer in Asteraceae ( *Bellis perennis* ), Poaceae (*Poa* spp.), Rosaceae ( *Potentilla anserina* ), Amaranthaceae (*Chenopodium* spp.), Brassicaceae (*Biscutella laevigata*), Plantaginaceae (*Plantago* spp.), and *Pimpinella* spp.

Among all the species found in the pastures, about 62 out of 114 showed a significant difference in relative abundance among the different areas. As reported in Figure S4, Busabella was characterized by the highest abundance of 12 species, including *Festuca rubra* , *Deschampsia caespitosa* , *Lolium arundinaceum* , *Phleum pratense* , and *Agrostis stolonifera* . Fosse had the highest abundance of 20 species, including *Sesleria caerulea* , *Erica carnea*, *Carex flacca* , *Prunella vulgaris* , and *Plantago media* . Piani della Vezzana showed the highest abundance of 15 species, including *Bistorta vivipara* , *Thymus pulegioides* , *Poa alpina* , *Lotus corniculatus* , and *Scabiosa velenovskyana*. Venegiota, finally, was characterized by the highest abundance of 14 species, including *Poa trivialis* , *Poa pratensis* , *Alchemilla vulgaris* , *Trifolium repens* , and *Taraxacum officinale* .

**FIGURE 10:**
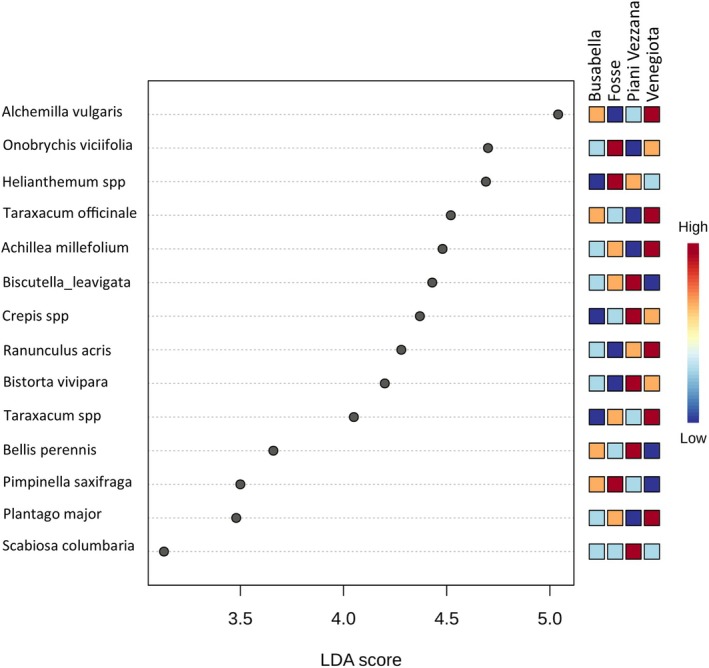
Results of the Linear Discriminant Analysis Effect Size (LEfSe) showing significant (*p* < 0.05) differences in the relative abundance of plant species found in marmot fecal samples from different areas. Species are arranged in descending order according to their LDA scores. Colors reflect the relative abundance in each macro‐area, where red indicates the highest abundance, followed by orange, light blue, and blue.

Among all 86 species considered typical according to incidence in marmot's diet, 14 showed a significant difference in abundance among different areas (Figure 10).

Busabella did not show the highest abundance in diet for any species, whereas Fosse was characterized by the highest abundance in the diet of *Onobrychis Viciifolia, Helianthemum* spp. and *Pimpinella saxifraga* . Piani della Vezzana was characterized by the highest abundance in the diet of *Biscutella laevigata*, *Crepis* spp., *Bistorta vivipara* , *Bellis perennis* and *Scabiosa columbaria* . Venegiota, finally, was characterized by the highest abundance in the diet of *Alchemilla vulgaris* , *Taraxacum officinale* , *Achillea millefolium* , *Ranunculus acris* , *Taraxacum* sp. and *Plantago major* .

**FIGURE 11:**
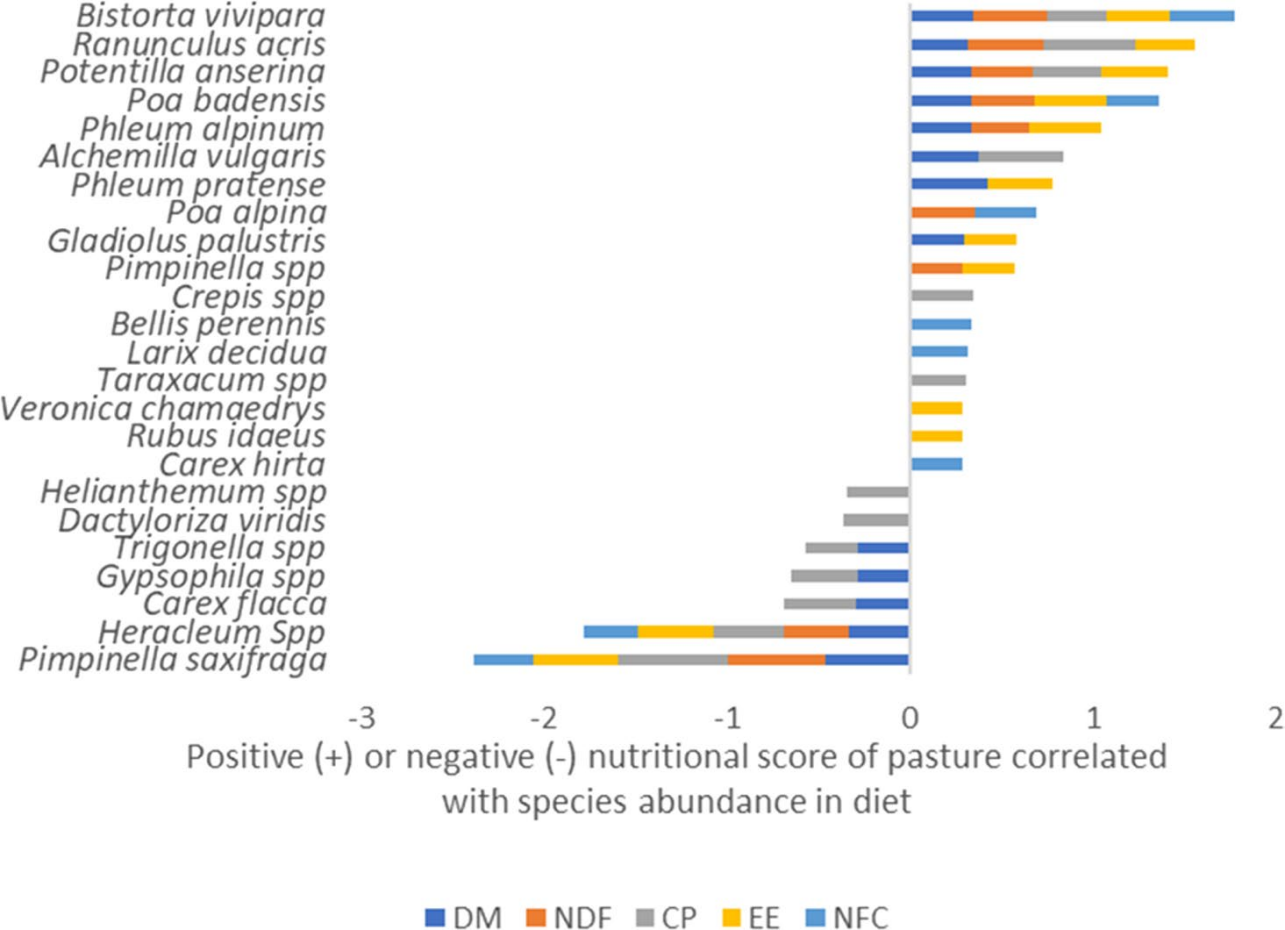
Plant species for which abundance in marmot diet was significantly correlated (Spearman, *p* < 0.05) with pasture overall nutritional score. DM and NDF were given negative scores when high and positive scores when low; conversely for CP, EE, and NFC. CP = crude protein; DM = dry matter; EE = ether extract; NDF = neutral detergent fiber; NFC = non‐fiber carbohydrates.

Significant (*p* < 0.05) Spearman correlation patterns between individual plant species abundance in fecal samples and nutritional characteristics of pastures were found for 24 species (Table S3). As shown in Figure 11, for 17 out of 24 species, the relative abundance in the marmot's diet is positively correlated with good nutritional quality proxies. *Bistorta vivipara* abundance was positively associated with all the nutritional proxies for a good quality pasture, represented by low DM and NDF and high CP, EE, and NFC. *Ranunculus acris, Potentilla anserina*, and *Poa badensis* were positively associated with 4 good pasture quality proxies out of 5, whereas other species were associated with a varying number of nutritional proxies. Seven species were balanced between positive and negative proxies, as, for example *Potentilla anserina* and *Ranunculus acris* , which were associated with low DM and high CP, but also with high NDF and low fat.

The remaining 7 species were positively correlated with low pasture quality proxies. Notably, *Pimpinella saxifraga* was positively associated with all the 5 proxies of low‐quality pasture, such as high DM and NDF, and low CP, EE, and NFC, followed by *Heracleum* sp., *Carex flacca* , and others (Figure 11).

The average nutrient composition of pastures in different months and macro‐areas is reported in Figure S5.

**FIGURE 12:**
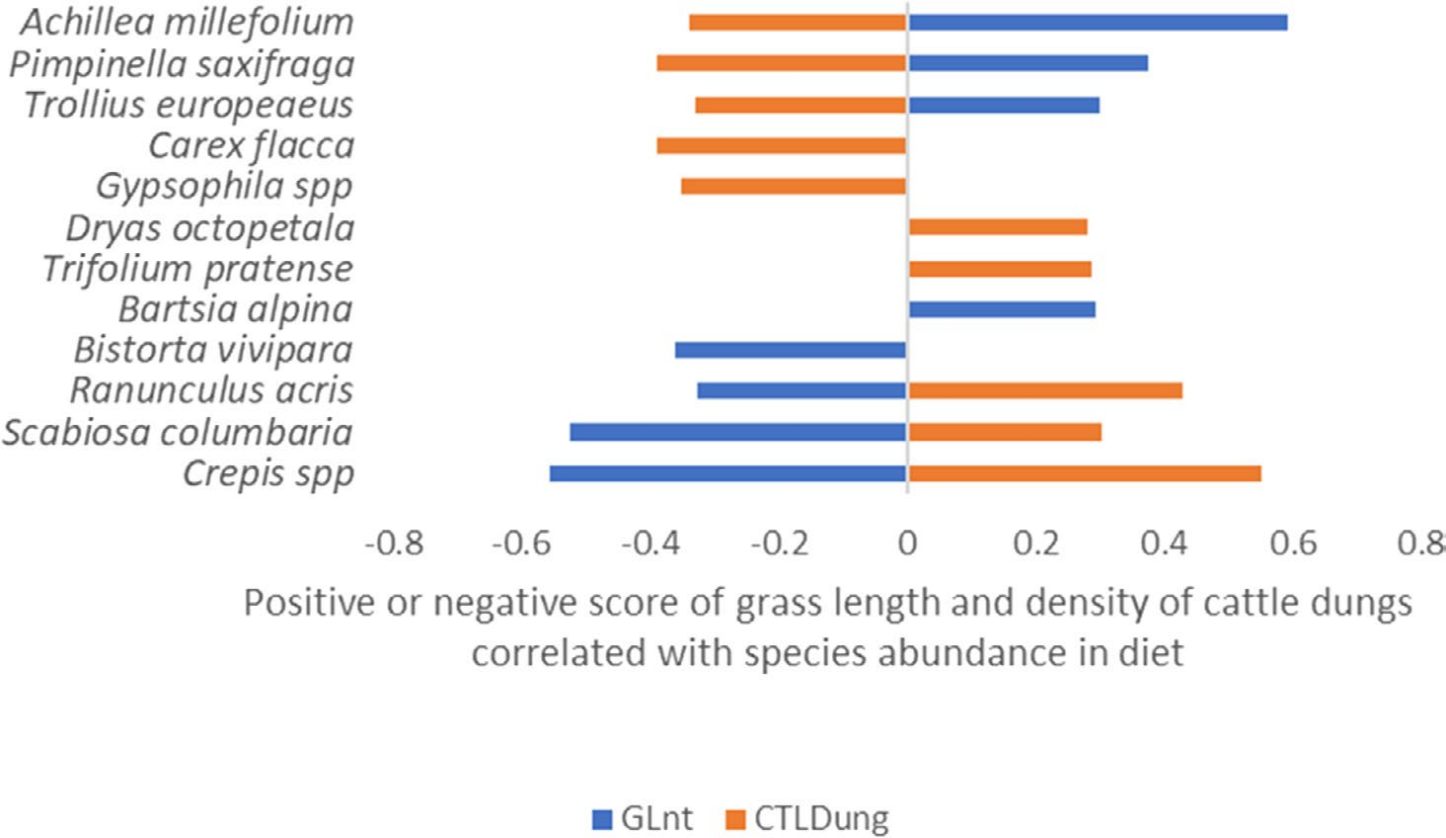
Plant species for which abundance in marmot diet was significantly correlated (Spearman, *p* < 0.05) with grass length (GLnt) and the density of cattle dung pats (CTLDung).

Twelve plant species were associated with grass length and/or the density of cattle dung pats (Figure 12). *Achillea millefolium* , *Pimpinella saxifraga* , and *Trollius europaeus* were positively correlated with tall herbage and a low density of cattle dung pats. Conversely, *Crepis* spp., *Ranunculus acris* , and *Scabiosa columbaria* were positively correlated with low herbage and a high density of dung pats. Other species were independently associated with either grass length or the density of dung pats (Figure 12).

As reported in Figure S6, 20 species show a significant (*p* < 0.05) Spearman correlation between their abundance in marmot diet and the concentration of certain fatty acid (FA) categories in the diet. Fourteen species were positively associated with the concentration of polyunsaturated fatty acids (PUFAs), including n‐6 (7 species) and n‐3 (8 species) FAs. Two species ( *Larix decidua* and *Bistorta vivipara* ) were negatively correlated with monounsaturated fatty acids (MUFAs). Two other species, *Heracleum* sp. and *Trifolium pratense* , were negatively associated with n‐3 FAs.

## 4 Discussion

This study helps in improving the understanding of the alpine marmot's dietary habits and foraging behavior. Through the application of DNA metabarcoding techniques, we have provided interesting insights into the alpine marmot's diet, identifying a wider range of plant species than previously reported. Our findings reveal that although alpine marmots exhibit a generalist feeding behavior, they also demonstrate selective foraging patterns influenced by plant nutrient composition, spatial variations in habitat, and seasonal changes. Furthermore, we have uncovered novel insights into the complex interplay between pasture nutritional quality, livestock grazing, and the marmots' dietary choices.

### 4.1 How Diverse is the Alpine Marmot's Diet, and To What Extent Do Marmots Selectively Forage on Specific Plant Species Relative to Their Availability in the Habitat?

The current study represents the first application of DNA metabarcoding to characterize the diet of the Alpine marmot. This study enabled the identification of plant species frequency and abundance, through the number of reads in the diet, at the species level, and the comparison, at the genus level, of the proportions of genera found in fecal samples and pastures. Previous investigations into the dietary habits of alpine marmots have relied on microscopic analysis of fecal material (Bassano et al. 1996; Massemin et al. 1996; Rudatis and Battisti 2006; Garin et al. 2008), which allowed for the quantification of plant families in the diet, whereas qualitative descriptions extended to the species level. The discrepancy in the techniques employed to identify plant species in fecal samples accounts for the substantially greater number of families (34), genera (70), and species (86) detected in the diet, as typical (Alberdi and Gilbert 2019), in our study compared to previous reports, which ranged from 11 species belonging primarily to 7 families (Bassano et al. 1996) to 32 species (Massemin et al. 1996) across 10–17 families (Rudatis and Battisti 2006; Garin et al. 2008). This finding is noteworthy, as the difference in the number of species observed in the pastures was considerably smaller, ranging from 158 (36 families) in our study to between 70 (Bassano et al. 1996; Massemin et al. 1996) and 28–30 families (Garin et al. 2008; Rudatis and Battisti 2006) compared to the variation among diets. Similar results have been observed in studies of other marmot species. Notably, Chovancová and Šoltésová (1988) and Karč (2006) identified between 9 and 11 plant species in the diet of the Tatra marmot ( *Marmota marmota latirostris* K.), whereas Aryal et al. (2015) detected 17 species in the scats of the Himalayan marmot ( *Marmota himalayana* H.) despite the presence of 78 plant species in the pastures utilized by these animals.

These results suggest a highly generalist feeding behavior in the Alpine marmot. The results of previous studies (Bassano et al. 1996; Massemin et al. 1996; Rudatis and Battisti 2006; Garin et al. 2008) in terms of diet breadth are more aligned with the number of typical species in the diet, on the basis of the abundance, which ranged on average from 7 to 11 species, according to the site (Table 2). *Alchemilla vulgaris* represented a dominant species in the fecal material of all the areas, followed by *Trifolium pratense* , *Carum carvi* , and *Heliantemum* spp., which were present as dominant species in the diet of two different areas. Notably, all dominant species (*q* = 2) on the basis of the abundance were forbs, with the exceptions of *Poa alpina* in Piani della Vezzana, which belongs to Poaceae, and *Picea abies* in Venegiota. Considering also the species included when *q* ≈ 1 (Table 2), the only other plants belonging to Poaceae in the diet are *Festuca rubra* in Busabella and Venegiota and *Phleum pratense* in Piani della Vezzana. This outcome confirms that forbs constitute the bulk of the alpine marmot's and other marmot species' diet (Table 2), as also reported by other authors (Bassano et al. 1996; Massemin et al. 1996; Garin et al. 2008; Ballová and Šibík 2015) and highlighted by the high presence of Rosaceae, Asteraceae, and Apiaceae in the diet in this study. However, typical species, on the basis of abundance, on average, accounted only for 62%–70% of the diet (Table 2), whereas the remaining 38%–30% was covered by a variety of many low abundant species. This is the reason why, to delve deeper into the relationship between diet and its drivers, the list of typical species on the basis of incidence (86 species) was chosen. Of these plant species, 17 were not detected in pasture (Table S2), probably because of their low abundance in pasture botanical composition that makes them difficult to be recorded using the Daget‐Poissonet method, which underestimates rare species (Gusmeroli 2012). Although it is well known that incidence data are less informative than abundance data (Alberdi and Gilbert 2019), in the present case, with few exceptions, the more abundant species also showed a high frequency (Figure 7).

Notwithstanding, the dietary composition of the alpine marmot could potentially encompass an even more diverse array of plant species than our findings indicate, as the present methodology employed the *trnL* gene region solely to detect and quantify the botanical taxa present in the diet, which, as elucidated by Goldberg et al. (2020), might have led to missing some species.

The sequence abundance of trnL genes in fecal matter is contingent upon the chloroplast density of the respective species and the digestibility of their plant tissues, thereby rendering the direct correlation between the fecal plant composition and the botanical composition of the ingested diet ambiguous (Palumbo et al. 2021). Nonetheless, as the abundance of plant species closely paralleled their frequency in the fecal samples (Table S2 and Figure 8), with few exceptions, this metric was judiciously employed to see whether the proportion of plant genera in the diet was similar, lower or higher than their proportion in the pasture, and we expressed this comparison as Jacob's index.

The high‐resolution genus‐level analysis of selectivity revealed that alpine marmots exhibit variable selection patterns for plant genera across different areas, likely because of differences in the availability of alternative genera, as proposed by Soininen et al. (2013). Among the dominant species in the diet (*q* = 2) of a given area (Table 2), those positively selected (positive Jacob's index) in that area, such as *Alchemilla* across all areas, *Carum* in Fosse and Venegiota, *Onobrychis* in Fosse, and *Biscutella* in Piani della Vezzana, likely possess superior nutritional characteristics or palatability. Conversely, genera that are dominant (*q* = 2) in an area but have a negative Jacob's index, such as *Trifolium* and *Poa* in Piani della Vezzana, are relatively abundant in the pasture (Figure S4) but less preferred compared to other genera, possibly because of lower nutritional value or the presence of undesirable secondary compounds (Carlsen and Fomsgaard 2008).

This selective feeding behavior corroborates the discrepancies observed between the proportions of plant families in the diet and those found in the pasture, as illustrated in Figure 4.

### 4.2 How Does the Alpine Marmot's Diet Vary Across Different Locations and Throughout The Summer Season?

According to our results, alpine marmots living in the study area showed some differences between the four areas in the dominant and typical plant species composing the diet (Table 2) and in the frequency of occurrence of some species (Figure 7). As shown in Figure S4, although different macro‐areas significantly differed in the relative abundance of more than half of the detected plant species (62 out of 114), only 14 differed in their abundance in marmot diets (Figure 10), highlighting that the differences between diets were not strictly linked to differences between pastures but were also due to a different selection occurring in the areas. Of the 14 species showing different abundance between areas, *Crepis* spp. in the diet followed the differences found in pastures; 8 species (*Helianthemum* spp., *Onobrychis viciifolia, Biscutella laevigata, Achillea millefolium, Bellis perennis, Pimpinella saxifraga, Taraxacum* sp., and *Scabiosa columbaria* ) had different proportions in diets from different macro‐areas, even though they did not differ in their proportions in pastures. The remaining 5 species ( *Alchemilla vulgaris* , *Bistorta vivipara* , *Taraxacum officinale*, *Ranunculus acris* , and *Plantago major* ) were differentially selected since their differences between areas in pastures did not match differences between areas in feces. Selection occurred not only for those species that differed in the diet but also for those that differed in their proportions in the pastures, yet had the same proportion in the diet. These findings align with those of Soininen et al. (2013) in voles, where different families and species within the plant functional groups (forbs and grasses) were consumed and selected to varying degrees. Although our findings demonstrate significant spatial variations in the alpine marmot's diet across different locations, it's equally important to consider how these dietary patterns change over time. The seasonal progression from late spring to late summer introduces another layer of complexity to the marmots' foraging behavior. By examining these temporal changes, we can gain a more comprehensive understanding of how Alpine marmots adapt their diet to both spatial and seasonal variations in their environment.

Regarding temporal variation, the botanical composition of the pasture did not exhibit consistent changes throughout the summer. The most substantial shift occurred between June and July, with less pronounced changes between July and August (Figure 10). Only *Agrostis stolonifera* and *Ranunculus montanus* demonstrated statistically significant changes in overall abundance. These findings suggest that although the relative abundance of most species remained stable throughout the summer, their phenological stages and nutritional profiles likely underwent changes. In fact, the phenological stage of available plants changes considerably over time, and the findings of Garin et al. (2008) indicate that the prevalence of vegetative, non‐reproductive plant growth exhibits a sharp decline between June and July, followed by a steady diminution as the season progresses. Conversely, the abundance of flowering and fruiting plants reaches its peak in July, only to virtually vanish by the arrival of September. As reported by other authors, different months contribute differently to the pasture's nutritional balance (Aryal et al. 2015), and overall, the nutrient content of herbaceous plants decreases toward the end of the growing season (Soininen et al. 2013). The protein content of most grasses decreases as the growing season progresses, but alpine marmots, like other rodents, frequently shift toward graminoid seeds during the late estival period, coinciding with the senescence of foliar and cauline structures in the majority of herbaceous vegetation (Rudatis and Battisti 2006; Goldberg et al. 2020).

As shown in Figure S5 and reported in the literature (Aryal et al. 2015; Goldberg et al. 2020), the protein, fat, and sugar balance of pastures is higher in June and more generally in late spring and early summer, whereas it generally decreases in July and August.

### 4.3 What is the Relationship Between the Alpine Marmot's Diet and Plant Nutrient Composition, and What Other Potential Factors Influence Their Dietary Choices?

Although earlier studies on food selection by mammalian herbivores have reported that the absolute and relative availability of a preferred food item may increase its consumption (Soininen et al. 2013; Goldberg et al. 2020), plant selection can also likely be related to the nutrients (Aryal et al. 2015), palatability, and digestibility present in different available plant parts for each species. Although various authors have argued that certain preferences, such as the marmots' predilection for forbs over graminoids, appear to be linked to differences in the nutritional quality of these two plant types (Carey and Moore 1986), no data have been presented to substantiate this claim. Garin et al. (2008) stated that forbs generally contain higher concentrations of nutrients (particularly phosphorus, calcium, sodium, and possibly protein) and lower levels of cell wall components (fiber), whereas Stallman and Holmes (2002) postulated that the choice of forbs over graminoids could be explained by the higher water content of forbs.

To our knowledge, only Aryal et al. (2015) have attempted to relate the feeding choices of marmots, in that case, the Himalayan marmot, to the nutritional composition of plants, finding a positive correlation between the available macronutrient content of food plants and their proportional representation in the diet. Similarly, it has been reported that tarbagans ( *Marmota sibirica* R.) avoid plants with high cellulose content, leading them to consume grasses and some herbs in the first half of the active season and primarily herbs in the second half (Ballová and Šibík 2015).

The present study is the first to demonstrate that in the alpine marmot, the abundance of certain plant species in the diet increases in accordance with the overall nutritional value of the pasture used by each family unit and over summer months. When pastures are rich in proteins, sugars, and low in dry matter (Figure 11), some species increase in abundance in the marmot diet. Conversely, when pastures are deficient in these nutrients and higher in fiber, the marmot diet becomes richer in other species.

Since marmots have a short active season to regain lean mass post‐hibernation and replenish fat deposits before the next hibernation period and they target high‐energy plants (Aryal et al. 2015), it is reasonable to assume that plant species more abundant in the diet when pasture nutritional quality is high are either those actively contributing to that quality (generally characterized by high sugar, protein, and fat content, with low fiber and dry matter) or those that only during that favorable period attain sufficient quality to be consumed. The optimal foraging theory predicts that when food is abundant, individuals are more likely to be selective and should choose higher‐quality foods (Goldberg et al. 2020). By cross‐referencing the results of genus selection with those of correlation to pasture quality, it becomes evident that *Alchemilla vulgaris* , *Crepis* spp., and *Taraxacum* spp. are positively selected, most likely because of their protein content (Figures 9 and 10). In contrast, *Bistorta vivipara* , *Potentilla anserina* , *Poa badensis*, and in some areas, *Ranunculus acris* and *Phleum pratense* , are likely consumed preferentially when the overall pasture quality is high, probably corresponding to nutritionally favorable phenological phases. Conversely, the species consumed more when pasture nutritional quality is poor likely represent plants that exhibit above‐average nutritional characteristics under such conditions, such as *Helianthemum* spp. and *Heracleum* sp. Alternatively, although not particularly nutritious on average, because of their phenological phase or intrinsic traits, they may represent the least unfavorable option, as observed with *Carex flacca* (Figures 9 and 10). As other small–to‐medium‐bodied hindgut fermenters, the alpine marmot relies on low‐fiber and high‐energy and protein foods (Hume et al. 1993; Wasserman and Chapman 2003). The significance of dietary protein for marmots is further corroborated by Holmes (1984), who documented a six‐fold increase in habitat utilization by hoary marmots ( *Marmota caligata* ) in specific grassland patches. This increased utilization was associated with an 11% elevation in the protein content of the vegetation in these areas.

Alpine marmots, like other rodents, are able to select their preferred forbs and grasses in spring and early summer but must incorporate less nutritious (but common) grasses as the summer progresses to ensure they obtain sufficient energy to survive overwinter hibernation. This aligns with findings on other rodents, where, in addition to the availability of a food item, the availability of alternative high‐quality food items may modify the plant composition of the diet, and food preferences are related to plant nutritional quality (Soininen et al. 2013). Collectively, these results demonstrate that alpine marmots exhibit flexible feeding ecology, primarily driven by pasture nutritional characteristics.

Although the nutritional composition of plants plays a crucial role in shaping the alpine marmot's diet, it is important to recognize that dietary choices are influenced by a complex interplay of factors. Our findings on the relationship between plant nutrient composition and marmot diet provide valuable insights, but they do not fully explain the observed patterns in foraging behavior. To gain a more comprehensive understanding, we must consider additional environmental and ecological factors that may influence the availability and quality of food resources for alpine marmots.

Seasonality is not the only factor involved in the change of pasture nutritional value, as both plant composition and the presence and degree of pasture grazing by livestock contribute to nutritional differences between areas in the same season (Figure S5). Grazing livestock affects the availability and phenological phases of plants, in particular by stimulating the production of new shoots in most plant species (Li et al. 2019). However, grazing stimulates the flowering phase in forbs having stems supporting the flowering parts (Li et al. 2019). Grasses possess a remarkable capacity for regeneration. Consequently, when subjected to grazing, the protein concentration and palatability of these forage species can persist at elevated levels, even as the growing season draws to a close (Radonjic et al. 2019). The possible impact of cattle grazing on the marmot diet is confirmed by the significant correlation between the abundance of some plants in the diet with herbage length and dung pats density, the proxy of cattle presence. High dung pats density and short herbage, which can be interpreted as the presence of grazing, favor the presence in the marmot's diet of *Crepis* spp., *Scabiosa columbaria*, *Ranunculus acris* , *Bistorta vivipara* (only short herbage), and *Trifolium pratense* (only high dung pats density). Notably, *Bistorta vivipara* and *Ranunculus acris* in the marmot's diet are also associated with low dry matter and high protein in the pasture, typical of young phenological phases, which can be favored by early summer (June) or grazing (Li et al. 2019). *Crepis* spp. in the diet, associated with high protein concentration in pastures (Figure 11), is present in the marmot diet from Venegiota and Piani della Vezzana (Figure 10), where cattle presence was high.

Conversely, *Pimpinella saxifraga* , *Gypsophila* spp., and *Carex flacca* are more present in the marmot's diet when pasture quality is low (Figure 11) and the presence of dung pats is low as well (Figure 12). A possible reason for this is that they could have a higher nutritional quality compared with the overall nutritional quality of the pasture in that specific context, where the fertilization effect of dung pats is low. Even though in literature, only a few data are available on the nutritional composition of individual alpine pasture plants, the high content in protein (16.7%–21.5%) of *Achillea millefolium* reported by Jayanegara et al. (2011), also showing a correlation between its presence in the diet and herbage height, and the low presence of dung pats corroborates this hypothesis.

These results can therefore confirm that there may be an interaction between livestock grazing and the composition of the marmot diet, which is also suggested for other rodents such as voles (Soininen et al. 2013). The effect of herbage height on the marmot diet likely extends beyond nutrient composition and possible interspecies competition between livestock and marmots and is inconsistent (Ballová and Šibík 2015). In contrast to the *M. marmota latirostris* subspecies, which exhibits a predilection for foraging in tall‐grass grasslands and lofty herbaceous plant communities belonging to the *Calamagrostion villosae* and *Trisetion fusci* alliances (Chovancová and Šoltésová 1988; Karč 2006), yellow‐bellied marmots demonstrate an inverse correlation between their foraging site preferences and areas characterized by dense, towering vegetation (Carey and Moore 1986). In habitats with tall vegetation, marmots must, in fact, increase their vigilance time when foraging and reduce their rate of food intake (Ballová and Šibík 2015).

Further drivers for the choice of plants to be included in the marmot diet can also involve the fatty acid content of different plant species and phenological phases. Polyunsaturated fatty acids, particularly linoleic and linolenic acid, are critical nutrients for some hibernating mammals (Ruf and Arnold 2008), including the alpine marmot, and some plant seeds or flowers contain high levels of polyunsaturated fatty acids (Garin et al. 2008). Elevated ratios of n‐6 to n‐3 polyunsaturated fatty acids (PUFAs) in cardiac myocyte membranes seem to confer protection against arrhythmia in the hibernating heart, a condition that in non‐hibernating hypothermic organisms arises from substantial elevations in cytosolic calcium levels (Ruf and Arnold 2008). Although n‐6 PUFA phospholipids are believed to stabilize cardiac function at low body temperatures, long‐chain (≥ C18) n‐3 PUFAs may enhance oxidative capacity (Arnold et al. 2011). The abundance of some plants in marmot diets (Figure S6) is positively correlated with PUFA and/or n‐3 and n‐6 PUFA at the pasture level, whereas others are more abundant when the concentration of PUFA in the pasture is lower and positively correlated with SFA. Although the functions of n‐3 and n‐6 polyunsaturated fatty acids (PUFAs) at the cellular membrane level are well‐established, their role as dietary constituents remains less certain. This is because some studies have demonstrated that substantial membrane remodeling in free‐ranging alpine marmots occurs largely independently of direct dietary intake (Arnold et al. 2011), and these animals maintain a consistent fatty acid composition in their white adipose tissue depots throughout the year, irrespective of the varying fatty acid profiles of their diet (Cochet et al. 1999).

### 4.4 Methodological Considerations

Although the present findings have contributed to broadening the understanding of the dynamic dietary choices exhibited by the alpine marmot and have highlighted the significance of nutritional drivers in shaping their foraging decisions, we acknowledge certain methodological constraints inherent to our approach. In contrast to micro histological techniques, DNA metabarcoding methodologies are fundamentally limited by their inability to discern the specific plant component (seeds, roots, or foliage) consumed by the animal, consequently precluding insights into potential temporal variations in the utilization of distinct plant parts contingent upon seasonal fluctuations. As it was not possible to account for relative differences in digestibility across various plant species or their respective components, it is plausible that the present assessment may have underestimated the marmots' preference for plants with elevated macronutrient content, particularly if such resources were represented by floral structures and fleshy fruits, which are usually highly digestible. Furthermore, the number of fecal samples available for analysis was constrained by the inherent challenges associated with locating and collecting such specimens in the field.

## 5 Conclusions

Our findings reveal that alpine marmots exhibit a remarkably diverse dietary repertoire, encompassing at least 86 plant species, which exhibit spatiotemporal variations. Moreover, the present results suggest that the proportional representation of these species in the marmots' dietary intake fluctuates in accordance with the nutritional composition at the pasture level and is not strictly dependent on their availability. This observation implies that marmots employ a strategic approach, combining non‐optimal food sources with variable yet complementary macronutrient ratios to compose a balanced diet. Furthermore, it was evident that the nutritional quality of pastures is subject to temporal variations, not only driven by seasonal changes but also influenced by livestock grazing activities. Although a more comprehensive understanding of seasonal dietary shifts and foraging preferences would necessitate detailed information on the nutrient content of different individual plant phenological phases, it is evident that in the face of a rapidly transforming landscape, a comprehensive understanding of the intricate relationship between seasonal dietary composition, resource availability, and foraging preferences of ecologically relevant species, such as the alpine marmot, is crucial. Such knowledge is imperative to inform and guide adaptive pasture management strategies tailored to target different botanical species throughout the annual cycle, particularly in light of the potential impact of anthropogenic disturbances on plant and animal communities.

## Author Contributions

**Giorgio Marchesini:** conceptualization (lead), data curation (equal), investigation (equal), methodology (equal), writing – original draft (lead). **Elena Basso:** data curation (equal), investigation (equal), methodology (equal), writing – review and editing (supporting). **Cristina Pornaro:** conceptualization (equal), data curation (equal), investigation (lead), writing – original draft (equal). **Piergiovanni Partel:** conceptualization (supporting), funding acquisition (lead), investigation (equal), writing – review and editing (supporting). **Gilberto Volcan:** investigation (equal), methodology (supporting), writing – review and editing (supporting). **Enrico Dorigatti:** investigation (equal), writing – review and editing (supporting). **Rudi Cassini:** methodology (lead), writing – review and editing (supporting). **Erica Marchiori:** investigation (equal), writing – review and editing (supporting). **Salvatore Raniolo:** formal analysis (equal), writing – review and editing (lead). **Massimo Pindo:** data curation (equal), investigation (equal), writing – review and editing (supporting). **Andrea Squartini:** data curation (equal), formal analysis (lead), writing – review and editing (supporting).

## Funding

This work was supported by the University of Padova, Department of Animal Medicine, Production and Health, DOR 2022, Paneveggio Pale di San Martino Natural Park, 2022.

## Conflicts of Interest

The authors declare no conflicts of interest.

## Supporting information


**Appendix S1:** ece373309‐sup‐0001‐AppendixS1.docx.
**Table S1:** List of plant families, genera, and species recorded in the pastures of the whole study area and their relative abundance (%), after filtering, in decreasing order by their abundance.
**Table S2:** Plant species frequency, average abundance (%), abundance standard deviation (SD), abundance coefficient of variation (CV), and presence or absence in pasture samples in overall alpine marmot's fecal specimens, shown in decreasing order by frequency.
**Table S3:** Spearman correlation coefficients (Corr) and significance (P) between plant species abundance in marmot diet and pasture nutrient composition. Only plants with a significant (p < 0.05) correlation with at least a nutrient were listed.
**Figure S1:** Rarefaction curves of plant species sampled in the pastures in different Areas. Labels of each line refer to each sampling transect.
**Figure S2:** Stacked bars graph on the species abundance of plant species found in pastures belonging to different areas under study. The list also includes the not‐assigned species category.
**Figure S3:** Stacked bars graph on the species abundance of plants found in alpine marmot's fecal samples in different areas filtered according the incidence‐based Hill number q1.
**Figure S4:** Results of the Linear Discriminant Analysis Effect Size (LEfSe) showing the significant (p < 0.05) differences in the relative abundance of plant species found in the pastures of different areas. Species are in decreasing order according to the LDA score. Colors reflect the relative abundance in the area, where red indicates the highest abundance, followed by orange, light blue and blue.
**Figure S5:** Average nutrient composition of Pastures per area and month. DM = dry matter (% wet weight); NDF = neutral detergent fiber (% DM); CP = crude protein (% DM); EE = ether ectract (% DM); NFC = non‐fiber carbohydrates (% DM).
**Figure S6:** Plant species for which abundance in the marmot diet was significantly correlated (Spearman, p < 0.05) with fatty acids categories in the pasture. SFA = saturated fatty acids; MUFA = Monounsaturated fatty acids; PUFA = Polyunsaturated fatty acids; n3 = Polyunsaturated fatty acids‐Omega 3; n6 = Polyunsaturated fatty acids‐Omega 6.

## Data Availability

Sequence reads, their metadata, and pasture data are deposited in the Research Data UNIPD repository (https://researchdata.cab.unipd.it/1692/). The statistical analyses were performed through Microbiome Analyst (https://www.microbiomeanalyst.ca/MicrobiomeAnalyst/). The R code to calculate Hill numbers is found in Alberdi and Gilbert (2019), https://doi.org/10.1111/1755‐0998.13014.
