# Supplementary material for: Unveiling Diet Preferences and Their Nutritional Drivers Through Metabarcoding: The Case of Alpine Marmot (Marmota marmota L.) in High Altitude Grazed Pastures
Source: Ecol Evol. 2026 Mar 22;16(3):e73309. doi: 10.1002/ece3.73309 (PMC13093508; doi:10.1002/ece3.73309)
Supplement: Supplementary file 1 — Appendix S1: ece373309‐sup‐0001‐AppendixS1.docx. Table S1: List of plant families, genera, and species recorded in the pastures of the whole study area and their relative abundance (%), after filtering, in decreasing order by their abundance. Table S2: Plant species frequency, average abundance (%), abundance standard deviation (SD), abundance coefficient of variation (CV), and presence or absence in pasture samples in overall alpine marmot's fecal specimens, shown in decreasing order by frequency. Table S3: Spearman correlation coefficients (Corr) and significance (P) between plant species abundance in marmot diet and pasture nutrient composition. Only plants with a significant (p < 0.05) correlation with at least a nutrient were listed. Figure S1: Rarefaction curves of plant species sampled in the pastures in different Areas. Labels of each line refer to each sampling transect. Figure S2: Stacked bars graph on the species abundance of plant species found in pastures belonging to different areas under study. The list also includes the not‐assigned species category. Figure S3: Stacked bars graph on the species abundance of plants found in alpine marmot's fecal samples in different areas filtered according the incidence‐based Hill number q1. Figure S4: Results of the Linear Discriminant Analysis Effect Size (LEfSe) showing the significant (p < 0.05) differences in the relative abundance of plant species found in the pastures of different areas. Species are in decreasing order according to the LDA score. Colors reflect the relative abundance in the area, where red indicates the highest abundance, followed by orange, light blue and blue. Figure S5: Average nutrient composition of Pastures per area and month. DM = dry matter (% wet weight); NDF = neutral detergent fiber (% DM); CP = crude protein (% DM); EE = ether ectract (% DM); NFC = non‐fiber carbohydrates (% DM). Figure S6: Plant species for which abundance in the marmot diet was significantly correlated (Spearman, [file ECE3-16-e73309-s001.docx]

Supporting information - Appendix

Table S1. List of plant families, genera and species recorded in the pastures of the whole study area and their relative abundance (%), after filtering, in decreasing order by their abundance.

| Family | Genus | *Species* |  | Overall abundance in pastures (%) |
| --- | --- | --- | --- | --- |
| Poaceae | *Festuca* | *Festuca rubra* |  | 8.78 |
| Fabaceae | *Trifolium* | *Trifolium repens* |  | 8.29 |
| Rosaceae | *Alchemilla* | *Alchemilla vulgaris* |  | 5.80 |
| Poaceae | *Deschampsia* | *Deschampsia caespitosa* |  | 5.15 |
| Apiacea | *Carum* | *Carum carvi* |  | 4.26 |
| Poaceae | *Poa* | *Poa alpina* |  | 4.20 |
| Polygonaceae | *Bistorta* | *Bistorta vivipara* |  | 4.01 |
| Poaceae | *Sesleria* | *Sesleria caerulea* |  | 3.55 |
| Poaceae | *Poa* | *Poa trivialis* |  | 3.39 |
| Poaceae | *Phleum* | *Phleum collare* |  | 3.30 |
| Poaceae | *Lolium* | *Lolium arrundinaceum* |  | 2.32 |
| Asteraceae | *Achillea* | *Achillea millefolium* |  | 2.13 |
| Ranunculaceae | *Ranunculus* | *Ranunculus montanus* |  | 1.81 |
| Poaceae | *Poa* | *Poa piatta* |  | 1.62 |
| Fabaceae | *Lotus* | *Lotus corniculatus* |  | 1.57 |
| Poaceae | *Agrostis* | *Agrostis stol* |  | 1.54 |
| Ranunculaceae | *Ranunculus* | *Ranunculus acris* |  | 1.51 |
| Cyperaceae | *Carex* | *Carex sempervirens* |  | 1.37 |
| Cyperaceae | *Carex* | *Carex flacca* |  | 1.32 |
| Lamiaceae | *Prunella* | *Prunella vulgaris* |  | 1.28 |
| Asteraceae | *Taraxacum* | *Taraxacum officinale* |  | 1.25 |
| Poaceae | *Phleum* | *Phleum pratense* |  | 1.20 |
| Poaceae | *Agrostis* | *Agrostis capillaris* |  | 1.16 |
| Asteraceae | *Senecio* | *Senecio alpino* |  | 1.11 |
| Asteraceae | *Crepis* | *Crepis aurea* |  | 1.05 |
| Lamiaceae | *Thymus* | *Thymus pulegioides* |  | 1.02 |
| Plantaginaceae | *Plantago* | *Plantago major* |  | 0.96 |
| Asteraceae | *Leontodon* | *Leontodon hispidus* |  | 0.94 |
| Plantaginaceae | *Plantago* | *Plantago media* |  | 0.90 |
| Lamiaceae | *Horminum* | *Horminum pyrenaicum* |  | 0.89 |
| Rosaceae | *Potentilla* | *Potentilla erecta* |  | 0.83 |
| Fabaceae | *Trifolium* | *Trifolium pratense* |  | 0.74 |
| Plantaginaceae | *Veronica* | *Veronica chamaedrys* |  | 0.73 |
| Poaceae | *Helictotricon* | *Helictotricon pratense* |  | 0.72 |
| Polygalaceae | *Rumex* | *Rumex alpino* |  | 0.70 |
| Asteraceae | *Cirsium* | *Cirsium acaulon* |  | 0.69 |
| Cyperaceae | *Carex* | *Carex capillaris* |  | 0.68 |
| Asteraceae | *Hieracium* | *Hieracium pilosella* |  | 0.61 |
| Asteraceae | *Homogine* | *Homogine alpina* |  | 0.56 |
| Poaceae | *Dactylis* | *Dactylis glomerata* |  | 0.55 |
| Poaceae | *Festuca* | *Festuca pratensis* |  | 0.53 |
| Plantaginaceae | *Globularia* | *Globularia cordifolia* |  | 0.51 |
| Cyperaceae | *Carex* | *Carex caryophylea* |  | 0.50 |
| Polygonaceae | *Rumex* | *Rumex acetosa* |  | 0.50 |
| Asteraceae | *Soldanella* | *Soldanella alpina* |  | 0.50 |
| Caprifoliaceae | *Scabiosa* | *Scabiosa velenovskyan* |  | 0.49 |
| Poaceae | *Agrostis* | *Agrostis gigantea* |  | 0.47 |
| Asteraceae | *Aposeris* | *Aposeris foetida* |  | 0.46 |
| Lamiaceae | *Betonica* | *Betonica alopecuros* |  | 0.46 |
| Fabaceae | *Hippocrepis* | *Hippocrepis comosa* |  | 0.43 |
| Poaceae | *Anthoxanthum* | *Anthoxanthum odoratum* |  | 0.41 |
| Poaceae | *Nardus* | *Nardus stricta* |  | 0.41 |
| Urticaceae | *Urtica* | *Urtica dioica* |  | 0.40 |
| Poaceae | *Briza* | *Briza media* |  | 0.38 |
| Cistaceae | *Helianthemum* | *Helianthemum alpestre* |  | 0.37 |
| Cyperaceae | *Carex* | *Carex pallescens* |  | 0.37 |
| Rosaceae | *Dryas* | *Dryas octopetala* |  | 0.35 |
| Cistaceae | *Helianthemum* | *Helianthemum nummularium* |  | 0.35 |
| Cyperaceae | *Carex* | *Carex ornithopoda* |  | 0.31 |
| Cyperaceae | *Blysmus* | *Blysmus compressus* |  | 0.28 |
| Rosaceae | *Potentilla* | *Potentilla cranzii* |  | 0.28 |
| Geraniaceae | *Geranium* | *Geranium pheum* |  | 0.27 |
| Apiacea | *Pimpinella* | *Pimpinella major* |  | 0.26 |
| Apiacea | *Heracleum* | *Heracleum frutto* |  | 0.23 |
| Cyperaceae | *Carex* | *Carex leporina* |  | 0.22 |
| Lamiaceae | *Galeopsis* | *Galeopsis tetrahit* |  | 0.22 |
| Fabaceae | *Astragalus* | *Astragalus monospessulanus* |  | 0.21 |
| Asteraceae | *Carlina* | *Carlina acaulis* |  | 0.19 |
| Tofieldiaceae | *Tofieldia* | *Tofieldia calyculata* |  | 0.19 |
| Polygalaceae | *Poligaloides* | *Poligaloides chamaebuxus* |  | 0.17 |
| Rubiaceae | *Galium* | *Galium album* |  | 0.15 |
| Lamiaceae | *Prunella* | *Prunella grandiflora* |  | 0.14 |
| Poaceae | *Festuca* | *Festuca rupicaprina* |  | 0.14 |
| Asteraceae | *Cardus* | *Cardus def* |  | 0.14 |
| Campanulaceae | *Campanula* | *Campanula shuezeri* |  | 0.13 |
| Fabaceae | *Anthillis* | *Anthillis vulneraria* |  | 0.13 |
| Asteraceae | *Scorzonera* | *Scorzonera aristata* |  | 0.13 |
| Caryophyllaceae | *Stellaria* | *Stellaria media* |  | 0.12 |
| Salicaceae | *Salix* | *Salix retusa* |  | 0.12 |
| Rubiaceae | *Galium* | *Galium pumilum* |  | 0.12 |
| Fabaceae | *Trifolium* | *Trifolium alpinum* |  | 0.11 |
| Asteraceae | *Centaurea* | *Centaurea nigrescens* |  | 0.11 |
| Clusiaceae | *Hypericum* | *Hypericum perforatum* |  | 0.11 |
| Cyperaceae | *Carex* | *Carex ferruginea* |  | 0.11 |
| Asteraceae | *Bellis* | *Bellis perennis* |  | 0.10 |
| Apiacea | *Antriscus* | *Antriscus sylvestris* |  | 0.10 |
| Ranunculaceae | *Trollius* | *Trollius europeus* |  | 0.09 |
| Ranunculaceae | *Ranunculus* | *Ranunculus hybridus* |  | 0.09 |
| Polygalaceae | *Poligala* | *Poligala vulgaris* |  | 0.09 |
| Thymelaeaceae | *Daphne* | *Daphne cneorum* |  | 0.08 |
| Caryophyllaceae | *Cerastium* | *Cerastium alpinum* |  | 0.08 |
| Gentianaceae | *Gentiana* | *Gentiana verna* |  | 0.08 |
| Violacee | *Viola* | *Viola biflora* |  | 0.08 |
| Poaceae | *Brachypodium* | *Brachypodium rupestre* |  | 0.07 |
| Caryophyllaceae | *Stellaria* | *Stellaria graminea* |  | 0.07 |
| Rosaceae | *Geum* | *Geum rivale* |  | 0.07 |
| Pinaceae | *Picea* | *Picea abies* |  | 0.07 |
| Cyperaceae | *Carex* | *Carex lepidocarpa* |  | 0.07 |
| Gentianaceae | *Gentiana* | *Gentiana bavarica* |  | 0.07 |
| Asteraceae | *Galinsoga* | *Galinsoga ciliata* |  | 0.07 |
| Ericaceae | *Vaccinium* | *Vaccinium mirtillus* |  | 0.06 |
| Poaceae | *Lolium* | *Lolium pratense* |  | 0.05 |
| Brassicaceae | *Biscutella* | *Biscutella laevigata* |  | 0.05 |
| Caryophyllaceae | *Arenaria* | *Arenaria serpillifolia* |  | 0.05 |
| Cyperaceae | *Carex* | *Carex atrata* |  | 0.04 |
| Asteraceae | *Petasites* | *Petasites alba* |  | 0.04 |
| Poaceae | *Poa* | *Poa pratense* |  | 0.04 |
| Asteraceae | *Cirsium* | *Cirsium spinosissimus* |  | 0.04 |
| Asteraceae | *Senecio* | *Senecio abrotanifolius* |  | 0.04 |
| Cyperaceae | *Luzula* | *Luzula campestris* |  | 0.04 |
| Cupressaceae | *Juniperus* | *Juniperus comunnis* |  | 0.03 |
| Cyperaceae | *Juncus* | *Juncus effusus* |  | 0.02 |
| Asteraceae | *Leucanthemum* | *Leucanthemum vulgare* |  | 0.02 |
| Poaceae | *Lolium* | *Lolium perenne* |  | 0.02 |

Table S2. Plant species frequency, average abundance (%), abundance standard deviation (SD), abundance coefficient of variation (CV) and presence or not in pasture samples in overall alpine marmot’s faecal specimens, shown in decreasing order by frequency.

| Species | Frequency | Average abundance (%) | SD | Present in pastures |
| --- | --- | --- | --- | --- |
| 1. **Refined list of species included by the Hill numbers based on incidence data** | | | | |
| ***Alchemilla vulgaris*** | 1 | 19.23 | 20.3 | Yes |
| ***Centaurea* sp.** | 1 | 2.61 | 2.74 | Yes |
| ***Taraxacum officinale*** | 1 | 5.43 | 8.1 | yes |
| ***Carum carvi*** | 0.98 | 10.54 | 17.11 | Yes |
| ***Leontodon hispidus*** | 0.98 | 1.91 | 3.18 | yes |
| ***Petasites albus*** | 0.98 | 1.56 | 2.95 | yes |
| ***Festuca rubra*** | 0.96 | 3.28 | 4.34 | yes |
| ***Poa alpina*** | 0.96 | 3.42 | 7.93 | yes |
| ***Prunella vulgaris*** | 0.96 | 2.05 | 5.87 | yes |
| ***Ranunculus acris*** | 0.94 | 2 | 5.05 | yes |
| ***Ranunculus repens*** | 0.94 | 0.7 | 1.14 | yes_nf |
| ***Rubus idaeus*** | 0.94 | 0.14 | 0.17 | yes_nf |
| ***Carlina acaulis*** | 0.9 | 0.39 | 0.72 | Yes |
| ***Rumex acetosa*** | 0.9 | 2.34 | 4.3 | yes |
| ***Trifolium pratense*** | 0.9 | 3.76 | 9.41 | yes |
| ***Bistorta vivipara*** | 0.88 | 1.38 | 2.22 | Yes |
| ***Achillea millefolium*** | 0.86 | 2.47 | 6.99 | Yes |
| ***Potentilla anserina*** | 0.84 | 0.1 | 0.16 | (no) |
| ***Trifolium repens*** | 0.84 | 1.83 | 3.8 | yes |
| ***Deschampsia cespitosa*** | 0.8 | 0.35 | 0.65 | yes |
| ***Helianthemum* spp.** | 0.8 | 5.58 | 15.75 | yes |
| ***Crepis* spp.** | 0.78 | 1.61 | 4.14 | yes |
| ***Phleum pratense*** | 0.78 | 2.14 | 4.29 | Yes |
| ***Heracleum* spp.** | 0.76 | 0.84 | 2.74 | yes |
| ***Lotus corniculatus*** | 0.76 | 1.89 | 4.12 | yes |
| ***Stellaria nemorum*** | 0.76 | 2.24 | 6.51 | (no) |
| ***Poa pratensis*** | 0.74 | 0.04 | 0.1 | yes |
| ***Pimpinella* sp.** | 0.7 | 0.03 | 0.09 | (no) |
| ***Taraxacum* spp.** | 0.7 | 0.96 | 1.6 | yes |
| ***Bellis perennis*** | 0.66 | 0.27 | 0.68 | Yes |
| ***Dryas octopetala*** | 0.66 | 0.02 | 0.05 | yes |
| ***Carex hirta*** | 0.62 | 0.19 | 0.54 | yes_nf |
| ***Agrostis stolonifera*** | 0.6 | 0.01 | 0.02 | Yes |
| ***Ranunculus* spp.** | 0.6 | 0.04 | 0.09 | yes |
| ***Senecio alpino*** | 0.6 | 0.44 | 1.97 | yes |
| ***Chenopodium* spp.** | 0.56 | 1.65 | 5.04 | yes_nf |
| ***Plantago lanceolata*** | 0.56 | 0.05 | 0.11 | (no) |
| ***Campanula rotundifolia*** | 0.54 | 0.3 | 0.87 | (no) |
| ***Crocus* spp.** | 0.54 | 0.76 | 1.83 | yes_nf |
| ***Plantago media*** | 0.54 | 0.29 | 0.7 | yes |
| ***Rumex alpinus*** | 0.54 | 0.01 | 0.01 | yes |
| ***Geum rivale*** | 0.52 | 0.35 | 1.3 | yes |
| ***Globularia cordifolia*** | 0.52 | 0.02 | 0.06 | yes |
| ***Dactylis glomerata*** | 0.5 | 0.02 | 0.12 | yes |
| ***Phleum alpinum*** | 0.5 | 0.01 | 0.02 | (no) |
| ***Pimpinella saxifraga*** | 0.5 | 0.19 | 0.51 | (no) |
| ***Biscutella leavigata*** | 0.48 | 1.11 | 4.75 | Yes |
| ***Plantago major*** | 0.48 | 0.21 | 0.59 | yes |
| ***Agrostis gigantea*** | 0.46 | 0.01 | 0.02 | Yes |
| ***Betula pendula*** | 0.44 | 0.05 | 0.12 | no |
| ***Picea abies*** | 0.42 | 0.79 | 4.42 | yes |
| ***Poa badensis*** | 0.42 | 0.01 | 0.02 | (no) |
| ***Silene vulgaris*** | 0.36 | 0.62 | 1.97 | (no) |
| ***Trollius europeaeus*** | 0.36 | 0.2 | 0.91 | yes |
| ***Vaccinium myrtillus*** | 0.36 | 1.07 | 4.76 | yes |
| ***Veronica chamaedrys*** | 0.36 | 0.05 | 0.14 | yes |
| ***Gladiolus palustris*** | 0.34 | 0.01 | 0.02 | no |
| ***Potentilla erecta*** | 0.34 | 0.01 | 0.03 | yes |
| ***Salix glauca*** | 0.34 | 0.15 | 0.4 | (no) |
| ***Urtica dioica*** | 0.32 | 0.08 | 0.37 | yes |
| ***Cardamine enneaphyllos*** | 0.3 | 0.02 | 0.1 | no |
| ***Ptychostomum amblyodon*** | 0.3 | 0.45 | 2.2 | no |
| ***Trigonella caerulea*** | 0.3 | 0.01 | 0.02 | no |
| ***Carex flacca*** | 0.28 | 0.06 | 0.28 | yes |
| ***Onobrychis viciifolia*** | 0.28 | 2.42 | 9.96 | no |
| ***Epilobium* spp.** | 0.26 | 0.22 | 1.16 | yes_nf |
| ***Galium odoratum*** | 0.26 | 0.01 | 0.03 | (no) |
| ***Geranium sylvaticum*** | 0.26 | 0.06 | 0.25 | yes_nf |
| ***Dactyloriza viridis*** | 0.24 | 0.06 | 0.24 | no |
| ***Kalmia procumbens*** | 0.24 | 0.08 | 0.37 | no |
| ***Larix decidua*** | 0.24 | 0.03 | 0.07 | no |
| ***Sorbus aucuparia*** | 0.24 | 1.1 | 5.26 | no |
| ***Luzula spicata*** | 0.22 | 0.05 | 0.18 | (no) |
| ***Pinus mugo*** | 0.22 | 0.1 | 0.34 | no |
| ***Avenella flexuosa*** | 0.2 | 0.04 | 0.22 | no |
| ***Dicranum bonjeanii*** | 0.2 | 0.2 | 0.93 | no |
| ***Trigonella* spp.** | 0.2 | 0.04 | 0.11 | no |
| ***Bartsia alpina*** | 0.18 | 0.01 | 0.05 | no |
| ***Bryum* spp.** | 0.18 | 0.16 | 0.8 | no |
| ***Soldanella alpina*** | 0.18 | 0.04 | 0.23 | yes |
| ***Stellaria graminea*** | 0.18 | 0.07 | 0.21 | yes |
| ***Gypsophila* spp*.*** | 0.16 | 0.09 | 0.52 | no |
| ***Scabiosa columbaria*** | 0.16 | 0.05 | 0.2 | (no) |
| ***Valeriana officinalis*** | 0.16 | 0.4 | 2.7 | no |
| ***Viola biflora*** | 0.16 | 0.09 | 0.43 | yes |
|  | | | | |
| 1. **Species not included by the Hill numbers based on incidence data** | | | | |
| *Myosotis scorpioides* | 0.24 | 0.04 | 0.18 | - |
| *Eritrichium nanum* | 0.14 | 0.01 | 0.05 | - |
| *Hypericum maculatum* | 0.14 | 0.08 | 0.37 | - |
| *Primula veris* | 0.14 | 0.06 | 0.28 | - |
| *Rhodiola rosea* | 0.14 | 1.38 | 9.49 | - |
| *Trachycarpus fortunei* | 0.14 | 0.03 | 0.11 | - |
| *Chamaenerion angustifolium* | 0.12 | 0.03 | 0.15 | - |
| *Luzula sudetica* | 0.12 | 0.16 | 0.89 | - |
| *Saxifraga oppositifolia* | 0.12 | 0.02 | 0.06 | - |
| *Betonica hirsuta* | 0.1 | 0.01 | 0.04 | - |
| *Ceratodon purpureus* | 0.1 | 0.01 | 0.07 | - |
| *Geranium pheum* | 0.1 | 0.04 | 0.27 | - |
| *Lloydia serotina* | 0.1 | 0.02 | 0.13 | - |
| *Poligala chamaebuxus* | 0.1 | 0.06 | 0.44 | - |
| *Barbula unguicolata* | 0.08 | 0.05 | 0.36 | - |
| *Convolvolus arvensis* | 0.08 | 0.01 | 0.04 | - |
| *Hedera helix* | 0.08 | 0 | 0 | - |
| *Oxyria digyna* | 0.08 | 0.01 | 0.04 | - |
| *Dicranella* spp. | 0.06 | 0.04 | 0.3 | - |
| *Gentianella engadinensis* | 0.06 | 0.01 | 0.06 | - |
| *Juniperus communis* | 0.06 | 0.02 | 0.07 | - |
| *Minuartia* spp. | 0.06 | 0.01 | 0.09 | - |
| *Oreojuncus trifidus* | 0.06 | 0.01 | 0.04 | - |
| *Pedicularis verticillata* | 0.06 | 0.06 | 0.41 | - |
| *Pohlia* spp. | 0.06 | 0.01 | 0.09 | - |
| *Primula farinosa* | 0.06 | 0.01 | 0.03 | - |
| *Silene acaulis* | 0.06 | 0.02 | 0.08 | - |
| *Vaccinium vitis-idaea* | 0.06 | 0.03 | 0.21 | - |
| *Veratrum album* | 0.06 | 0.01 | 0.07 | - |
| *Dichodontium flavescens* | 0.04 | 0.16 | 1.13 | - |
| *Melanpyrum sylvaticum* | 0.04 | 0.04 | 0.3 | - |
| *Prunus armeniaca* | 0.04 | 0.04 | 0.25 | - |
| *Schistidium* spp. | 0.04 | 0.02 | 0.13 | - |
| *Setaria verticillata* | 0.04 | 0.1 | 0.73 | - |

Species in bold = included in the refined list based on Hill numbers (incidence-based); No = Not detected in pasture; (no) = Genus detected in pasture, but not the specific species; yes = Detected in pasture; yes_nf = Detected in pasture when including less frequent, non-filtered species. - = Species for which the correspondence in the pasture was not considered, as they were not included by the Hill numbers based on incidence data.


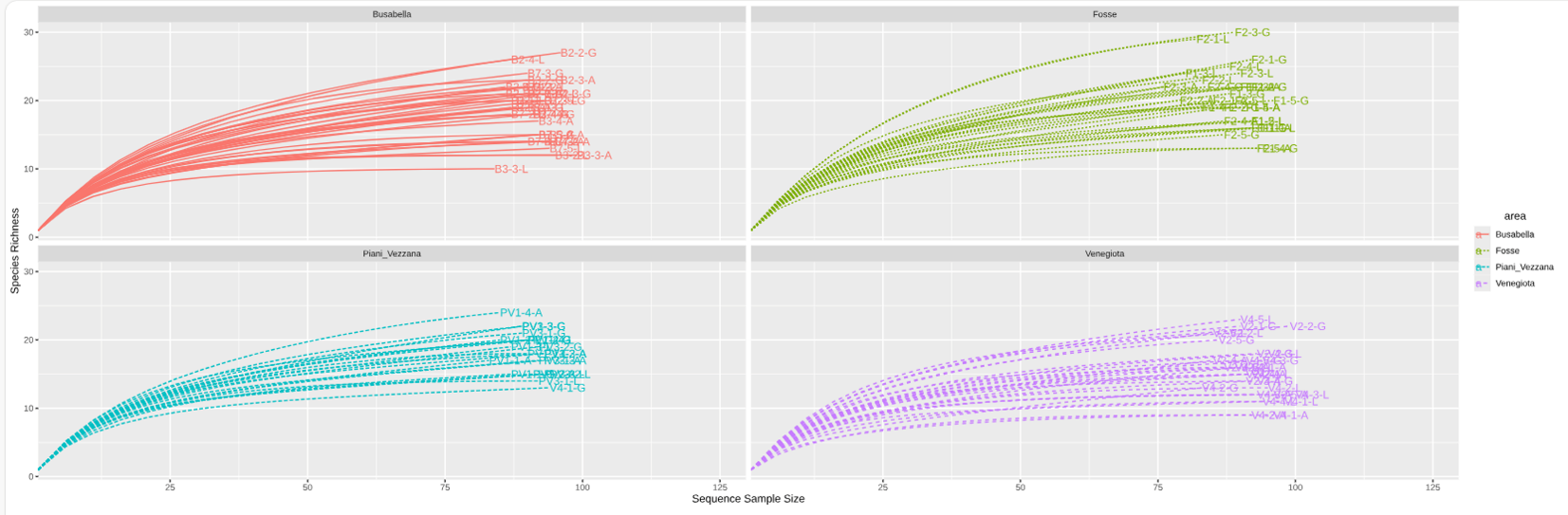


Figure S1. Rarefaction curves of plant species sampled in the pastures in different Areas. Lables of each line refer to each sampling transect.


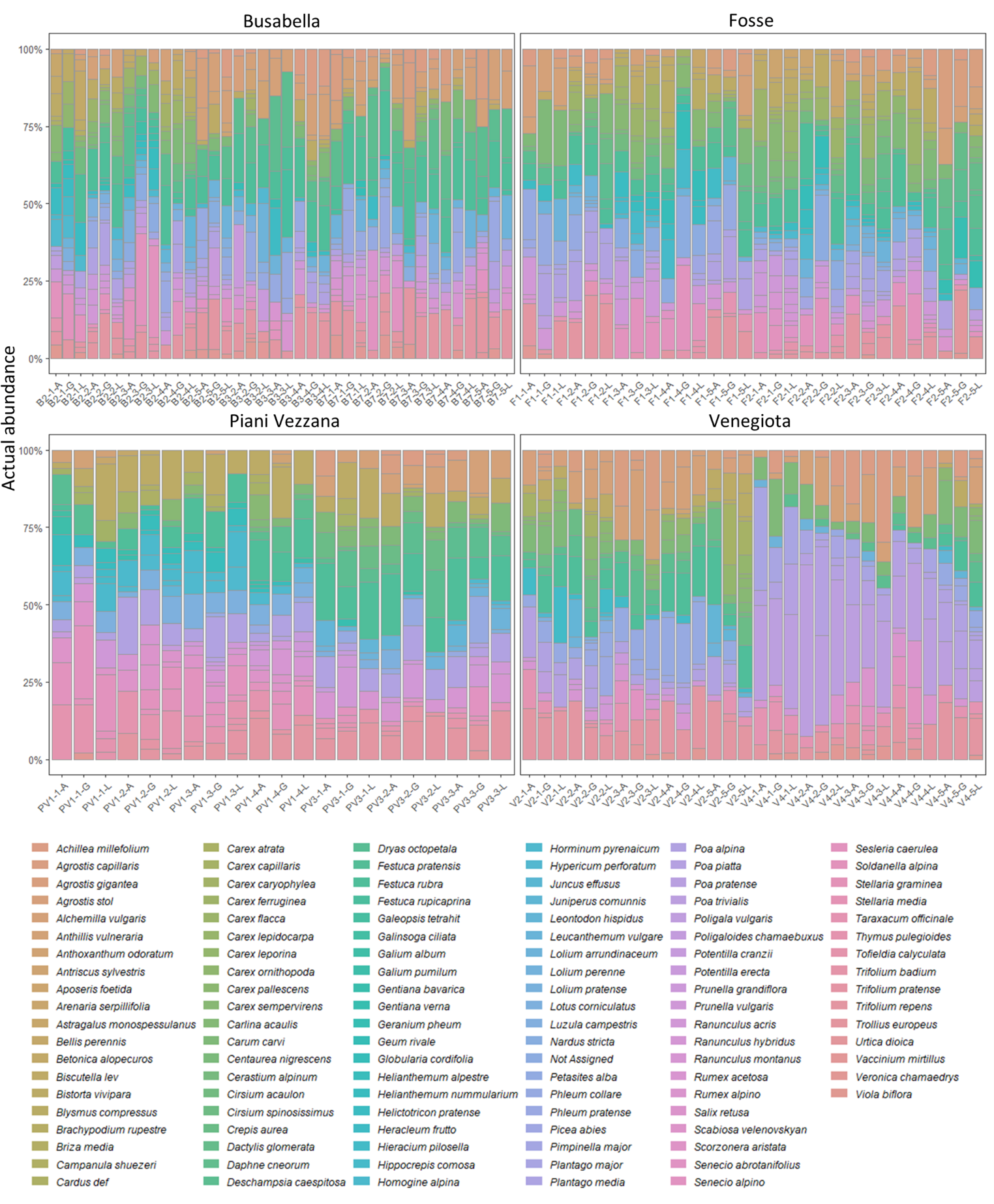


Figure S2. Stacked bars graph on the species abundance of plant species found in pastures belonging to different areas under study. The list includes also the not assigned species category.


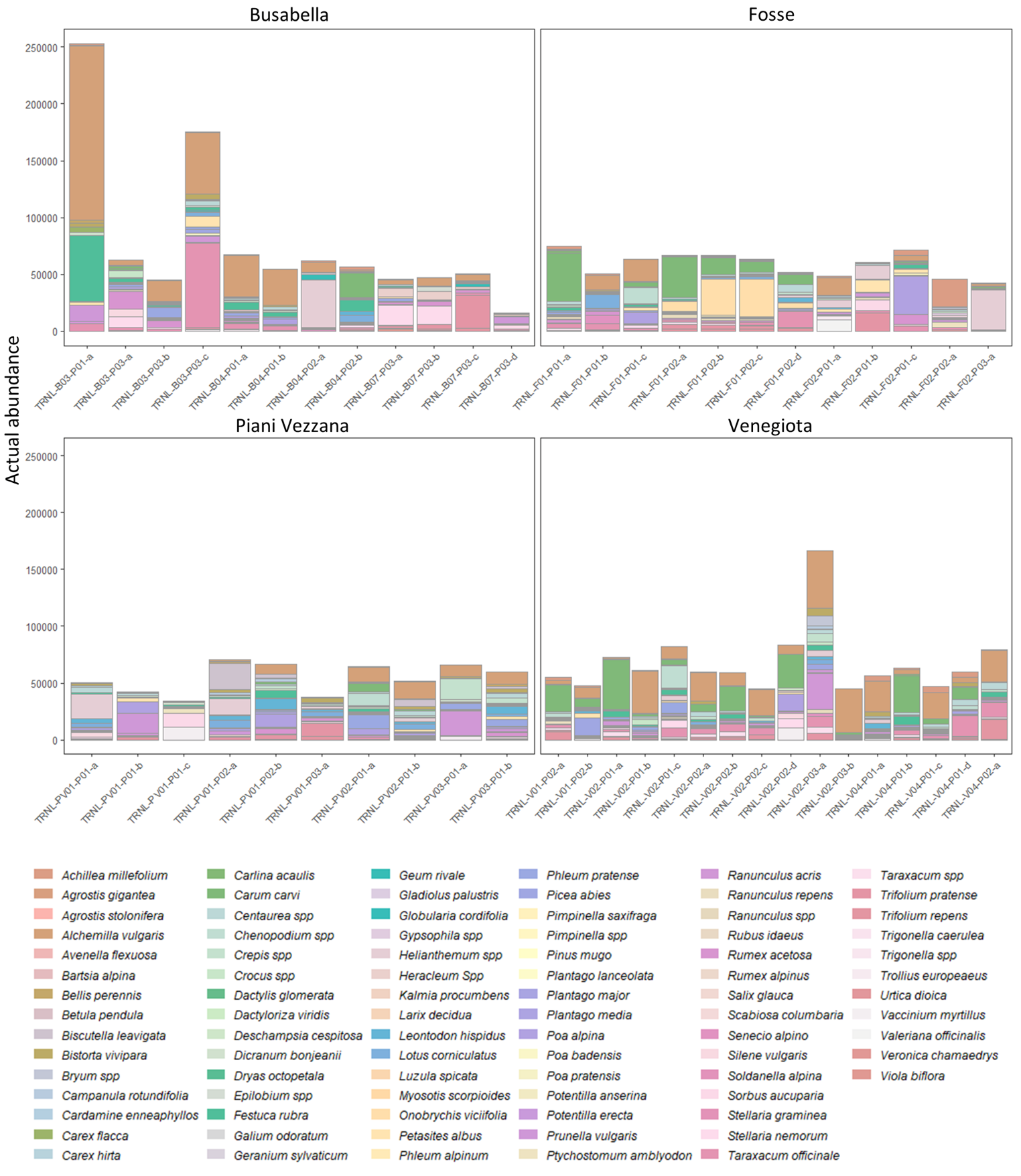


Figure S3. Stacked bars graph on the species abundance of plants found in alpine marmot ‘s faecal samples in different areas filtered according the incidence-based Hill number q^1^.


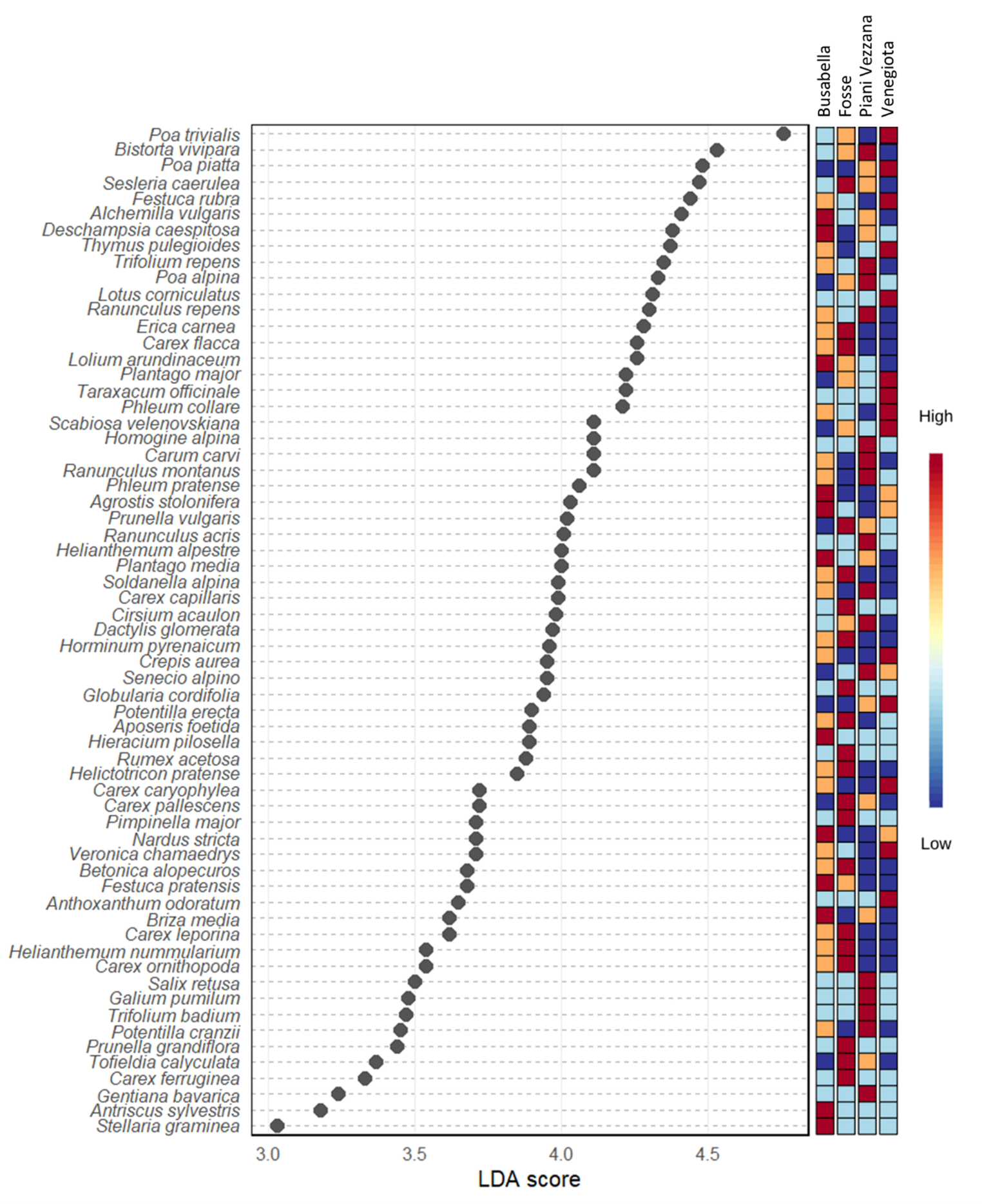


Figure S4. Results of the Linear Discriminant Analysis Effect Size (LEfSe) showing the significant (P < 0.05) differences in the relative abundance of plant species found in the pastures of different areas. Species are in decreasing order according to the LDA score. Colours reflect the relative abundance in the area, where red indicates the highest abundance, followed by orange, light blue and blue.

Table S3. Spearman correlation coefficients (Corr) and significance (*P*) between plant species abundance in marmot diet and pasture nutrient composition. Only plants with a significant (p < 0.05) correlation with at least a nutrient were listed.

| Species | DM | | CP | | EE | | NDF | | NFC | |
| --- | --- | --- | --- | --- | --- | --- | --- | --- | --- | --- |
|  | Corr | P | Corr | P | Corr | P | Corr | P | Corr | P |
| *Alchemilla vulgaris* | -0.377 | 0.007 | 0.458 | 0.001 | 0.259 | 0.069 | -0.236 | 0.099 | 0.034 | 0.813 |
| *Bellis perennis* | -0.133 | 0.358 | -0.073 | 0.613 | 0.083 | 0.565 | -0.202 | 0.160 | 0.335 | 0.017 |
| *Bistorta vivipara* | -0.349 | 0.013 | 0.319 | 0.024 | 0.351 | 0.012 | -0.401 | 0.004 | 0.352 | 0.012 |
| *Carex flacca* | 0.299 | 0.035 | -0.395 | 0.005 | -0.192 | 0.182 | 0.245 | 0.087 | -0.052 | 0.722 |
| *Carex hirta* | -0.152 | 0.292 | 0.235 | 0.101 | 0.236 | 0.099 | -0.250 | 0.080 | 0.285 | 0.045 |
| *Crepis spp* | 0.004 | 0.977 | 0.342 | 0.015 | 0.107 | 0.459 | -0.240 | 0.093 | 0.170 | 0.238 |
| *Dactyloriza viridis* | 0.259 | 0.070 | -0.369 | 0.008 | -0.183 | 0.203 | 0.269 | 0.059 | -0.114 | 0.430 |
| *Gladiolus palustris* | -0.292 | 0.039 | 0.120 | 0.407 | 0.290 | 0.041 | -0.276 | 0.053 | 0.221 | 0.123 |
| *Gypsophila spp* | 0.285 | 0.045 | -0.361 | 0.010 | -0.204 | 0.155 | 0.171 | 0.235 | -0.059 | 0.684 |
| *Helianthemum spp* | 0.215 | 0.133 | -0.349 | 0.013 | -0.069 | 0.632 | 0.065 | 0.655 | 0.129 | 0.373 |
| *Heracleum Spp* | 0.336 | 0.017 | -0.380 | 0.007 | -0.418 | 0.003 | 0.354 | 0.012 | -0.294 | 0.038 |
| *Larix decidua* | -0.149 | 0.302 | 0.076 | 0.601 | 0.168 | 0.243 | -0.260 | 0.068 | 0.314 | 0.026 |
| *Phleum alpinum* | -0.334 | 0.018 | 0.262 | 0.066 | 0.395 | 0.005 | -0.311 | 0.028 | 0.218 | 0.129 |
| *Phleum pratense* | -0.422 | 0.002 | 0.211 | 0.141 | 0.356 | 0.011 | -0.267 | 0.061 | 0.239 | 0.095 |
| *Pimpinella saxifraga* | 0.462 | 0.001 | -0.602 | 0.000 | -0.459 | 0.001 | 0.532 | 0.000 | -0.333 | 0.018 |
| *Pimpinella spp* | -0.131 | 0.366 | 0.269 | 0.059 | 0.284 | 0.046 | -0.289 | 0.042 | 0.254 | 0.075 |
| *Poa alpina* | -0.213 | 0.138 | 0.089 | 0.538 | 0.259 | 0.069 | -0.354 | 0.012 | 0.335 | 0.017 |
| *Poa badensis* | -0.331 | 0.019 | 0.248 | 0.083 | 0.392 | 0.005 | -0.346 | 0.014 | 0.287 | 0.043 |
| *Potentilla anserina* | -0.335 | 0.017 | 0.378 | 0.007 | 0.358 | 0.011 | -0.334 | 0.018 | 0.226 | 0.115 |
| *Ranunculus acris* | -0.317 | 0.025 | 0.506 | 0.000 | 0.330 | 0.019 | -0.407 | 0.003 | 0.253 | 0.077 |
| *Rubus idaeus* | -0.211 | 0.140 | 0.231 | 0.106 | 0.286 | 0.044 | -0.222 | 0.121 | 0.158 | 0.274 |
| *Taraxacum spp* | 0.020 | 0.890 | 0.305 | 0.031 | 0.174 | 0.228 | -0.141 | 0.330 | 0.087 | 0.548 |
| *Trigonella spp* | 0.284 | 0.046 | -0.286 | 0.044 | 0.023 | 0.875 | 0.185 | 0.198 | -0.080 | 0.580 |
| *Veronica chamaedrys* | -0.199 | 0.165 | 0.191 | 0.185 | 0.290 | 0.041 | -0.211 | 0.141 | 0.080 | 0.579 |

DM = dry matter; NDF = neutral detergent fiber; CP = crude protein; EE = ether ectract; NFC = non-fibre carbohydrates. Significant (*p* < 0.05) positive correlations are highlighted in green, whereas significant negative correlations are highlighted in red.


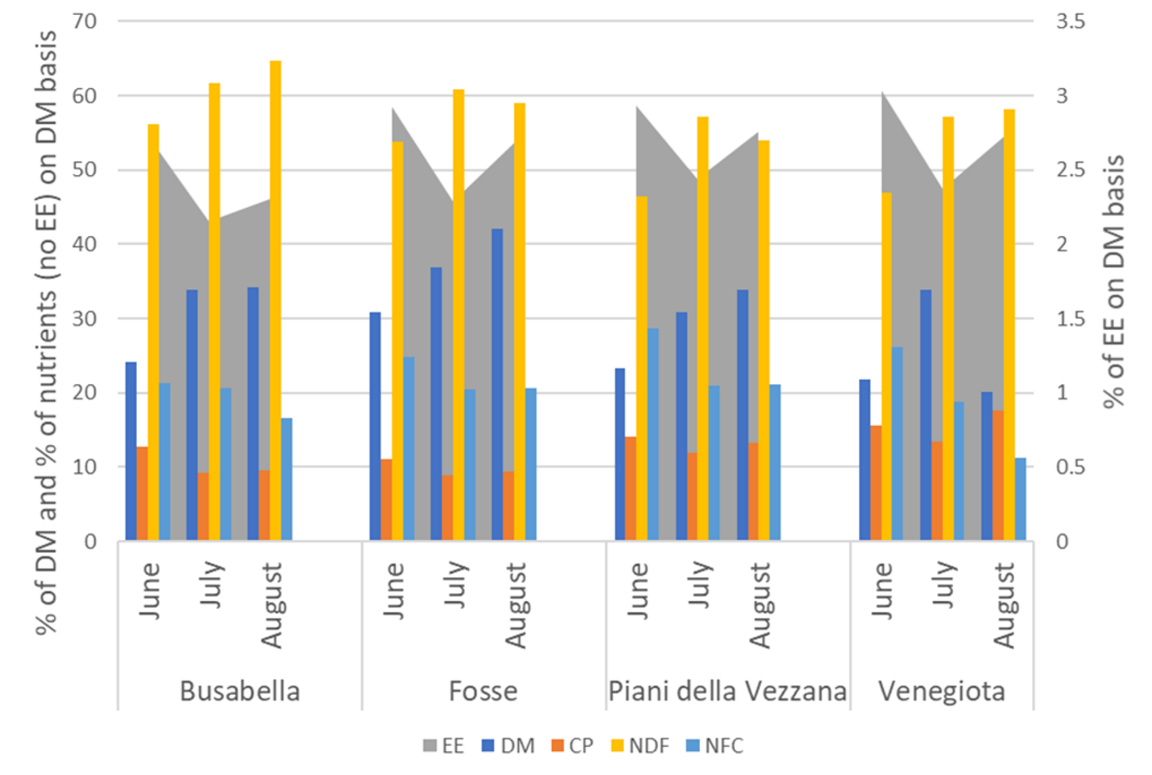


Figure S5. Average nutrient composition of Pastures per area and month. DM = dry matter (% wet weight); NDF = neutral detergent fiber (% DM); CP = crude protein (% DM); EE = ether ectract (% DM); NFC = non-fibre carbohydrates (% DM).


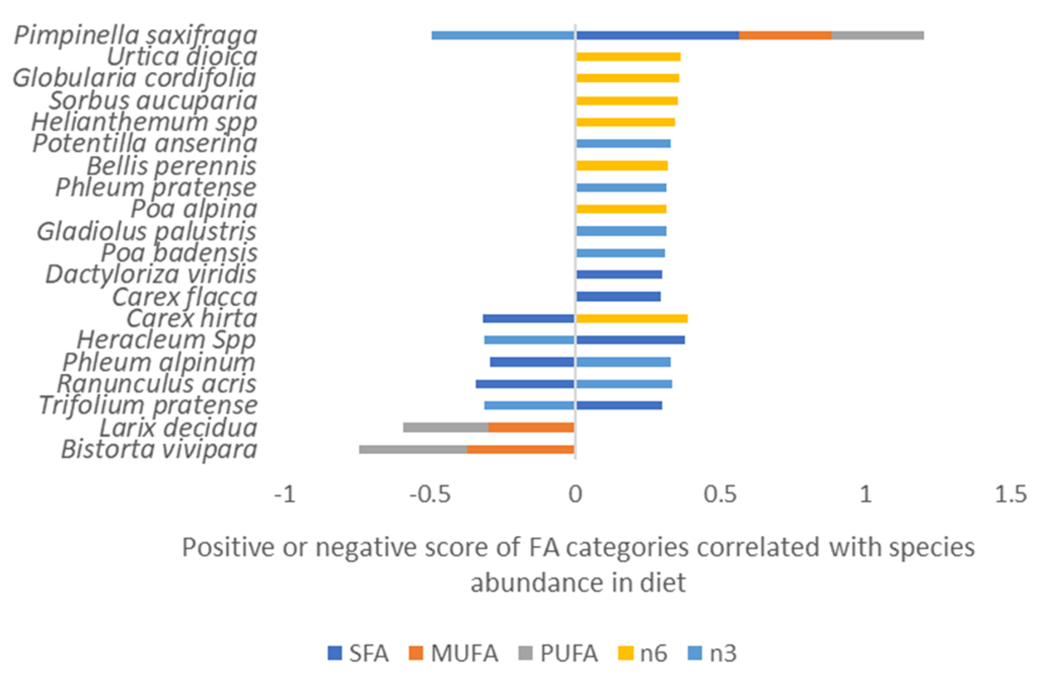


Figure S6. Plant species for which abundance in marmot diet was significantly correlated (Spearman, p < 0.05) with fatty acids categories in the pasture. SFA = saturated fatty acids; MUFA = Monounsaturated fatty acids; PUFA = Polyunsaturated fatty acids; n3 = Polyunsaturated fatty acids-Omega 3; n6 = Polyunsaturated fatty acids-Omega 6.
